# *Eupolybothrus cavernicolus* Komerički & Stoev sp. n. (Chilopoda: Lithobiomorpha: Lithobiidae): the first eukaryotic species description combining transcriptomic, DNA barcoding and micro-CT imaging data

**DOI:** 10.3897/BDJ.1.e1013

**Published:** 2013-10-28

**Authors:** Pavel Stoev, Ana Komerički, Nesrine Akkari, Shanlin Liu, Xin Zhou, Alexander M. Weigand, Jeroen Hostens, Christopher I. Hunter, Scott C. Edmunds, David Porco, Marzio Zapparoli, Teodor Georgiev, Daniel Mietchen, David Roberts, Sarah Faulwetter, Vincent Smith, Lyubomir Penev

**Affiliations:** † National Museum of Natural History, Sofia, Bulgaria; ‡ Pensoft Publishers, Sofia, Bulgaria; § Croatian Biospeleological Society, Zagreb, Croatia; | Natural History Museum of Denmark, University of Copenhagen, Copenhagen, Denmark; ¶ China National GeneBank, BGI-Shenzhen, Shenzhen, China; # Goethe-University, Institute for Ecology, Evolution and Diversity, Frankfurt am Main, Germany; †† Bruker microCT, Kontich, Belgium; ‡‡ GigaScience, BGI HK Ltd., Tai Po, Hong Kong, China; §§ Université de Rouen - Laboratoire ECODIV, Mont Saint Aignan Cedex, France; || Università degli Studi della Tuscia, Department for Innovation in Biological, Agro-food and Forest systems (DIBAF), Viterbo, Italy; ¶¶ Museum für Naturkunde – Leibniz-Institut für Evolutions- und Biodiversitätsforschung, Berlin, Germany; ## The Natural History Museum, London, United Kingdom; ††† National and Kapodestrian University of Athens, Athens, Greece; ‡‡‡ Hellenic Centre for Marine Research, Heraklion, Greece; §§§ Institute of Biodiversity & Ecosystem Research - Bulgarian Academy of Sciences and Pensoft Publishers, Sofia, Bulgaria

**Keywords:** Cybertaxonomy, gene sequence data, micro-CT, data integration, molecular systematics, caves, Croatia, biospeleology

## Abstract

We demonstrate how a classical taxonomic description of a new species can be enhanced by applying new generation molecular methods, and novel computing and imaging technologies. A cave-dwelling centipede, *Eupolybothrus cavernicolus* Komerički & Stoev sp. n. (Chilopoda: Lithobiomorpha: Lithobiidae), found in a remote karst region in Knin, Croatia, is the first eukaryotic species for which, in addition to the traditional morphological description, we provide a fully sequenced transcriptome, a DNA barcode, detailed anatomical X-ray microtomography (micro-CT) scans, and a movie of the living specimen to document important traits of its ex-situ behaviour. By employing micro-CT scanning in a new species for the first time, we create a high-resolution morphological and anatomical dataset that allows virtual reconstructions of the specimen and subsequent interactive manipulation to test the recently introduced ‘cybertype’ notion. In addition, the transcriptome was recorded with a total of 67,785 scaffolds, having an average length of 812 bp and N50 of 1,448 bp (see GigaDB). Subsequent annotation of 22,866 scaffolds was conducted by tracing homologs against current available databases, including Nr, SwissProt and COG. This pilot project illustrates a workflow of producing, storing, publishing and disseminating large data sets associated with a description of a new taxon. All data have been deposited in publicly accessible repositories, such as GigaScience GigaDB, NCBI, BOLD, Morphbank and Morphosource, and the respective open licenses used ensure their accessibility and re-usability.

## Introduction

While 13,494 new animal species were discovered by taxonomists in 2012 (Index of Organism Names), animal diversity on the planet continues to decline with unprecedented speed (Balmford et al. 2003). Changes and intensification of land use, habitat destruction, human population growth, pollution, exploitation of marine resources and climate change are among the major factors that lead to biodiversity impoverishment, and for the first time in human history, the rate of species extinction may exceed that of species discovery (Wheeler et al. 2012). The rapid pace of extermination has forced taxonomists to speed up the process of biodiversity investigation. The ‘turbo-taxonomy’ approach, combining molecular data, concise morphological descriptions, and digital imaging, has recently been introduced (Butcher et al. 2012, Riedel et al. 2013a) as one solution for the global loss of taxonomic expertise, part of the problem generally referred to as ‘taxonomic impediment’ (Wägele et al. 2011). Accelerated ‘pipeline’ descriptions of 178 new species of parasitic wasps (Butcher et al. 2012) and 101 new species of *Trigonopterus* weevils (Riedel et al. 2013b) were recently used to exemplify the concept.

Here, we present a more holistic approach to taxonomic descriptions. It is exemplified through a new cave centipede, *Eupolybothrus cavernicolus* Komerički & Stoev sp. n., recently discovered by biospeleologists in Croatia. To the best of our knowledge, this is the first time the description of a new eukaryotic species has been enhanced with rich genomic and morphological data, including a fully sequenced transcriptome, DNA barcodes, detailed X-ray micro-computed tomography scanning (micro-CT), and a video of a living specimen showing behavioural features. In this increasingly data-driven era, a further aim of this study is to set a new standard for handling, management and publishing of various data types. It is essential that data are easily accessible to researchers in every field of science, and able to be integrated from many sources, to tackle complex and novel scientific hypotheses. Rapid advances and increasing throughput of technologies such as phenotyping, genome-scale sequencing and meta-barcoding are now producing huge volumes of data, but there has been a lag in efforts to curate, present, harmonise and integrate these data to make them more accessible and re-usable for the community. Furthermore, by employing micro-CT scanning we test for the first time in a new taxon the recently introduced ‘cybertype’ notion (Faulwetter et al. 2013) of high-resolution virtual morphological and anatomical data libraries allowing reconstruction and interactive manipulation of type specimens.

To respond to the increasing interest in exposing and publishing biodiversity data (see e.g., Penev et al. 2011, Costello et al. 2013, Drew et al. 2013) and following the recent developments in open access data publishing (Smith et al. 2013) we also propose a novel workflow in the *Biodiversity Data Journal* of producing, storing, evaluating, publishing and disseminating complex data sets. The large-scale data handling, management and storage was provided by the GigaScience GigaDB database (see Stoev et al. 2013), with transcriptomic and annotation data made publicly available to the most stringent metadata standards in INSDC (NCBI/EMBL/DDBJ) databases, GigaDB and the relevant datatype specific repositories.

### The study group

The subfamily Ethopolyinae Chamberlin, 1915 is known to comprise some of the largest lithobiomorphs in the world, with several species reaching 45-50 mm in length. At present, the subfamily includes four more or less well defined genera: *Bothropolys* Wood, 1862 with around 40 species from North America and East Asia; *Archethopolys* Chamberlin, 1925 with three species from the southwestern USA, *Zygethopolys* Chamberlin, 1925 with four species from western Canada and the USA, and *Eupolybothrus* Verhoeff, 1907 with 23 valid and 15 doubtful species and subspecies assigned to seven subgenera ranging from Southern Europe and North Africa to the Near and Middle East, including the largest Mediterranean islands Corsica, Sardinia, Sicily, Crete and Cyprus (Zapparoli and Edgecombe 2006, Zapparoli and Edgecombe 2011). The genus *Eupolybothrus* exhibits the highest species diversity in the Italian and Balkan peninsulas (Zapparoli 2003), where a number of cave-dwelling species have restricted distribution ranges. A further 66 species-level taxa proposed in *Eupolybothrus* are currently considered to be junior synonyms, although their taxonomic status might change in the light of future taxonomic and molecular studies. The exact placement of genus *Ethopolys* Chamberlin, 1912, with twelve species in two subgenera from western Canada and the USA is uncertain, being treated in contemporary literature as either a synonym of *Bothropolys* (Zapparoli and Edgecombe 2006, Zapparoli and Edgecombe 2011) or a valid genus (Mercurio 2010).

While some species of *Eupolybothrus* and the genus itself have been treated recently in several publications (see e.g., Eason 1970, Zapparoli 1984, Zapparoli 1995, Zapparoli 1998, Zapparoli and Edgecombe 2006, Iorio 2008, Stoev et al. 2010), the other three genera, with few exceptions (e.g., Matic 1974, Ma et al. 2008, Ma et al. 2009, Ma 2012) have remained out of the scope of contemporary studies. Nevertheless, it is also far from being fully revised, as a number of problems are still in need of modern scrutiny. These mainly concern: 1) a high number of vaguely described or/and poorly known species and subspecies, mostly from the Balkans and Anatolia, known only from their original description; 2) an outdated subgeneric classification that lacks any phylogenetic framework; and 3) a high number of cryptic taxa in the *Eupolybothrus nudicornis* (Gervais, 1837), *Eupolybothrus litoralis* (L. Koch, 1867) and *Eupolybothrus tridentinus* (Fanzago, 1874) species-groups, as recently revealed by application of DNA barcoding (Porco et al. 2011, Komerički et al. 2012). Further, Stoev et al. (2010) found high interspecific divergence values (20.8% mean value) between two closely related *Eupolybothrus* species in another barcoding study with mitochondrial Cytochrome C Oxidase subunit I (COI). Two other studies (Edgecombe and Giribet 2003, Spelda et al. 2011) contributed genomic data by analysing DNA barcodes for *Eupolybothrus fasciatus* and *Eupolybothrus tridentinus* from Italy and Germany, respectively. The present study is part of an ongoing revision of the subfamily Ethopolyinae (Stoev et al. 2010, Porco et al. 2011, Komerički et al. 2012).

## Materials and methods

### Collected material and morphological study

The present study is based on eight specimens of *Eupolybothrus cavernicolus* Komerički & Stoev sp. n. belonging to the Croatian Biospeleological Society (CBSS), the National Museum of Natural History, Sofia (NMNHS) and the Natural History Museum of Denmark (ZMUC). The specimens were preserved in ethanol (70 or 96%) or RNA*later* (Qiagen, USA). The morphological study of the new species was performed at NMNHS and CBSSS with a Zeiss microscope. For scanning electron microscopy (performed at ZMUC), parts of the specimens were cleaned by ultrasonification, transferred to 96% ethanol and then to acetone, air-dried, mounted on adhesive electrical tape attached to aluminium stubs, coated with platinum/palladium and studied in a JEOL JSM-6335F scanning electron microscope. Images were edited in Adobe Lightroom 4.3 and Adobe Photoshop CS 5. All morphological images have been deposited in Morphbank. Terminology for external anatomy follows Bonato et al. (2010).

### Molecular experiments and sequencing

**DNA barcode sequencing**

DNA extraction was conducted in the the Canadian Centre for DNA Barcoding, Guelph on complete animals or part of the leg of the specimens preserved in 96% ethanol. Standard protocols of the Canadian Centre for DNA Barcoding were used for both DNA extraction and amplification. All specimen data are stored in the Barcode of Life Data System (BOLD) online database and are available also in the dataset DS-EUPCAV (http://dx.doi.org/10.5883/DS-EUPCAV), where they are linked to the respective Barcode Index Numbers clusters. This dataset contains sequences from ten species: *Eupolybothrus cavernicolus* Komerički & Stoev sp. n., *Eupolybothrus leostygis* (Verhoeff, 1899), *Eupolybothrus obrovensis* (Verhoeff, 1930), *Eupolybothrus grossipes* (CL Koch, 1847), *Eupolybothrus gloriastygis* (Absolon, 1916), *Eupolybothrus nudicornis*, *Eupolybothrus litoralis*, *Eupolybothrus kahfi*, *Eupolybothrus transsylvanicus* (Latzel, 1882) and *Eupolybothrus tridentinus*. In addition, all sequences were registered in GenBank (accession numbers KF715038-KF715064, HM065042-HM065044, HQ941581-HQ941585, JN269950, JN269951, JQ350447, JQ350449), one sequence of *Eupolybothrus fasciatus* (Newport, 1845) was recovered from GenBank (accession number AY214420). Two sequences from two *Lithobius species* were included as outgroups: *Lithobius austriacus* (Verhoeff, 1937) (MYFAB442-11) and *Lithobius crassipes* L. Koch, 1962 (MYFAB443-11). The final dataset comprises 39 sequences. Molecular delimitation of species was achieved by the implementation of the Automatic Barcoding Gap Discovery (ABGD) procedure as described in Puillandre et al. (2012) and by the reversed Statistical Parsimony (SP) approach as suggested by Hart and Sunday (2007). A Neighbor-Joining (NJ) tree was built for visualization.

For the ABGD method, we tested various model combinations to cross-check the obtained results: relative gap with (X) ranging from 0.05 to 1.5, minimal intraspecific distance (Pmin) of 0.001 and maximal intraspecific distance (Pmax) ranging from 0.02 to 0.11. Pmin and Pmax refer to the genetic distance area where the barcoding gap should be detected, whereas X defines the width of the gap. Distance calculation was based on the Kimura-2-parameter model and a transition/transversion ratio of 2.0. The method was performed in 100 steps. Statistical Parsimony networks for the delineation of species were reconstructed on the basis of 95% statistical confidence (i.e. connection probability) using the program TCS 1.21 (Clement et al. 2000). The NJ-topology was calculated in MEGA 5.0 (Tamura et al. 2011) using the K2P-model under the pairwise-deletion option and 1000 bootstrap replicates. Intra- and interspecific genetic K2P-distances were calculated in MEGA 5.0 as well.

**Transcriptome sequencing**

One entire adult male specimen of *Eupolybothrus cavernicolus* Komerički & Stoev sp. n. was crushed and preserved in liquid RNA*later* (Qiagen, USA) immediately after being captured. To extract total RNA, TRIzol reagent (Invitrogen, USA) was used according to the manufacturer’s instructions. Messenger RNA (mRNA) was isolated from total RNA using a Dynabeads mRNA Purification Kit (Invitrogen, USA). The mRNA was fragmented and transcribed into first-strand cDNA using SuperScript™II Reverse Transcriptase (Invitrogen, USA) and N6 primer (IDT). RNase H (Invitrogen, USA) and DNA polymerase I (Invitrogen, Shanghai China; New England BioLabs) were subsequently applied to synthesize the second-strand of the cDNA. The double-stranded cDNA then underwent end-repair, a single ‘A’ base addition, adapter ligation, and size selection, indexed and PCR amplified to construct a library. The extracted cDNA was utilised for library construction with an insert size of 250 bp. Finally, the library was sequenced on the Illumina HiSeq2000 sequencing platform (Illumina, Inc., San Diego, California, USA) at BGI-Shenzhen using a 150bp pair-end strategy to generate a total of 2.5 Gb raw reads. Illumina HCS1.5.15.1 + RTA1.13.48.0 were applied to generate a “bcl” file which was then downloaded to local computers. Secondly, the “bcl” file was converted to qseq format using BclConverter-1.9.0-11-03-08. Finally, we separated individual sample data from multiplexed machine runs based on the specific barcode primer sequences, and converted the file format to fastq.

### Micro-CT scanning

The micro-CT scanning of one adult female specimen was performed at Bruker microCT, Kontich, Belgium, using a SkyScan 1172 system with the following settings: 40kV, 0.43° rotation step, acquiring 839 projection images from 360° with a pixel size of 8µm. Prior to scanning, the sample was dehydrated in graded ethanol: 50%, 70%, 90%, 100%, for 2 hours in total, and then transferred to HMDS (hexamethyldisilasane) for 2 hours, and air dried. Reconstruction was done with the SkyScan software NRecon, using a modified Feldkamp algorithm, and adjusting for beam hardening and applying ring artefact correction resulting in 3865 cross sections in. bmp format, with image size 2000x2000 pixels. The video of 3D volume renderings was created with CTVox, using the flight recorder function, and saved as an AVI (Audio Video Interface) file. The obtained data were processed through a transfer function where the different voxels with different grey value were (or weren't) made opaque and where the color was assigned to a certain grey value. The image stack is stored in GigaDB (Stoev et al. 2013) under a Creative Commons CC0 public domain waiver. The only software used was CTVox, a viewing software, not analysis software (although you could argue that viewing the images is also a way of analyzing them).

### Abbreviations

T– Tergite, TT– Tergites, Legs: L– left, R– right; Plectrotaxy table: Cx– coxa, Tr– trochanter, Pf– prefemur, F– femur, T– tibia, a, m, p stand for spines in respectively, anterior, medial and posterior position.

## Taxon treatments

### Eupolybothrus cavernicolus

Komerički & Stoev, 2013 sp. n.

urn:lsid:zoobank.org:act:6F9A6F3C-687A-436A-9497-70596584678C

http://www.ebi.ac.uk/ena/data/view/ERP003841

http://www.ebi.ac.uk/arrayexpress/experiments/E-MTAB-1859

http://dx.doi.org/10.5524/100063

KF715043

KF715049

KF715050

KF715059

http://dx.doi.org/10.5883/DS-EUPCAV

999021821&tsn=true

http://morphosource.org/index.php/Detail/SpecimenDetail/Show/specimen_id/514

#### Materials

- **Occurrence:** recordedBy: M. Lukić; individualCount: 1; sex: male; lifeStage: adult; **Location:** country: Croatia; stateProvince: Knin; locality: NP Krka, village Kistanje, Hydroelectric power plant Miljacka, cave Miljacka II; verbatimElevation: 115 m; verbatimLatitude: 44°00'01.1''N; verbatimLongitude: 16°00'58.5''E; **Event:** samplingProtocol: hand collected under clay sediment; eventDate: 9 February 2013; **Record Level:** institutionCode: CBSS; collectionCode: CHP536
- **Occurrence:** recordedBy: A. Komerički; individualCount: 1; sex: female; lifeStage: adult; **Location:** country: Croatia; stateProvince: Knin; locality: NP Krka, village Kistanje, Hydroelectric power plant Miljacka, cave Miljacka II; verbatimElevation: 115 m; verbatimLatitude: 44°00'01.1''N; verbatimLongitude: 16°00'58.5''E; **Event:** samplingProtocol: hand collected under lump of clay; eventDate: 18 October 2012; **Record Level:** institutionCode: CBSS; collectionCode: CHP517
- **Occurrence:** recordedBy: M. Lukić; individualCount: 1; sex: male; lifeStage: adult; **Location:** country: Croatia; stateProvince: Knin; verbatimLocality: NP Krka, village Kistanje, Hydroelectric power plant Miljacka, cave Miljacka II; verbatimElevation: 115 m; verbatimLatitude: 44°00'01.1''N; verbatimLongitude: 16°00'58.5''E; **Event:** samplingProtocol: hand collected; eventDate: 9 February 2013; **Record Level:** institutionCode: BGI
- **Occurrence:** recordedBy: H. Cvitanović & A. Komerički; individualCount: 1; sex: female; lifeStage: adult; **Location:** country: Croatia; stateProvince: Knin; verbatimLocality: NP Krka, village Kistanje, Hydroelectric power plant Miljacka, cave Miljacka II; verbatimElevation: 115 m; verbatimLatitude: 44°00'01.1''N; verbatimLongitude: 16°00'58.5''E; **Event:** samplingProtocol: hand collected; eventDate: 18 October 2012; **Record Level:** institutionCode: NMNHS; collectionCode: NMNHS-CHILOPODA-1/2013
- **Occurrence:** recordedBy: A. Kirin & A. Komerički; individualCount: 1; sex: female; lifeStage: adult; **Location:** country: Croatia; stateProvince: Knin; verbatimLocality: NP Krka, village Kistanje, Hydroelectric power plant Miljacka, cave Miljacka II; verbatimElevation: 115 m; verbatimLatitude: 44°00'01.1''N; verbatimLongitude: 16°00'58.5''E; **Event:** samplingProtocol: hand collected; eventDate: 4 May 2010; **Record Level:** institutionCode: ZMUC; collectionCode: zmuc00029439
- **Occurrence:** recordedBy: M. Lukić; individualCount: 1; sex: male; lifeStage: adult; **Location:** country: Croatia; stateProvince: Knin; verbatimLocality: NP Krka, village Kistanje, cave Miljacka IV (= Špilja kod mlina na Miljacki); verbatimElevation: 115 m; verbatimLatitude: 44°00'12.8''N; verbatimLongitude: 16°01'08.8''E; **Event:** samplingProtocol: hand collected; eventDate: 4 May 2010; **Record Level:** institutionCode: ZMUC; collectionCode: zmuc00029440
- **Occurrence:** recordedBy: A. Kirin; individualCount: 1; sex: female; lifeStage: subadult; **Location:** country: Croatia; stateProvince: Knin; verbatimLocality: NP Krka, village Kistanje, Hydroelectric power plant Miljacka, cave Miljacka II; verbatimElevation: 115 m; verbatimLatitude: 44°00'01.1''N; verbatimLongitude: 16°00'58.5''E; **Event:** samplingProtocol: hand collected; eventDate: 4 May 2010; **Record Level:** institutionCode: CBSS; collectionCode: CHP420
- **Occurrence:** recordedBy: A. Komerički; individualCount: 1; sex: damaged female; lifeStage: subadult; **Location:** country: Croatia; stateProvince: Knin; verbatimLocality: NP Krka, village Kistanje, cave Miljacka IV (= Špilja kod mlina na Miljacki); verbatimElevation: 115 m; verbatimLatitude: 44°00'12.8''N; verbatimLongitude: 16°01'08.8''E; **Event:** samplingProtocol: hand collected; eventDate: 4 May 2010; **Record Level:** institutionCode: CBSS; collectionCode: CHP416
- **Occurrence:** recordedBy: A. Komerički; individualCount: 1; sex: damaged male; lifeStage: adult; **Location:** country: Croatia; stateProvince: Knin; verbatimLocality: NP Krka, village Kistanje, Hydroelectric power plant Miljacka, cave Miljacka II; verbatimElevation: 115 m; verbatimLatitude: 44°00'01.1''N; verbatimLongitude: 16°00'58.5''E; **Event:** samplingProtocol: hand collected; eventDate: 21 October 2012; **Record Level:** institutionCode: CBSS; collectionCode: CHP552

#### Description

##### Description of holotype

**Body length:** *approx.* 30 mm (measured from anterior margin of cephalic plate to posterior margin of telson); leg 15 – 22.6 mm long, or 75% length of body.

**Figure 1.**
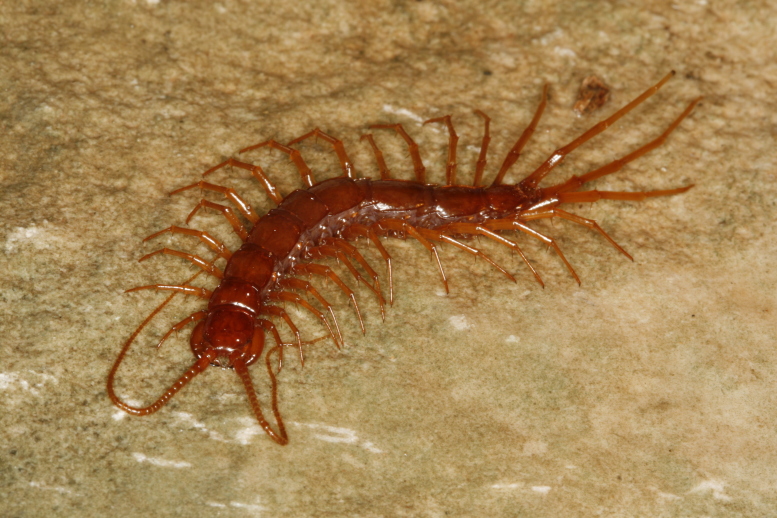
Habitus of *Eupolybothrus cavernicolus* Komerički & Stoev sp. n., male paratype, *ex situ.*

**Color:** uniformly yellow-brownish to chestnut, margins of cephalic plate slightly darker than inner parts (Fig. 1).

**Head:** cephalic plate broader than long (4.0 x 3.6 mm, respectively), as wide as T1 (Fig. 2a); surface smooth, with several minute scattered pits, setae generally absent, except for a few emerging from the marginal ridge (above ocelli) and on the median sulcus. Cephalic median sulcus contributing to biconvex anterior margin, marginal ridge with a median thickening; posterior margin straight or slightly concave; transverse suture situated at about 1/3^rd^ of anterior edge; posterior limbs of transverse suture visible, connecting basal antennal article with anterior part of ocellar area. **Ocelli:** 1+14 blackish, irregular in shape, in 3-4 rows, outermost first seriate ocellus largest, ocelli of the middle two rows medium-sized, those of inferior row smallest (Fig. 2b). **Tömösváry’s organ:** moderately large (as large as a medium ocellus), oval, situated on subtriangular sclerotisation below the inferiormost row of seriate ocelli (Fig. 2b). **Clypeus**: with a cluster of 25-30 setae situated on the apex and near the lateral margin (Fig. 3a). **Antennae:** right antenna composed of 71 articles, left antenna damaged after 61^st^ article; slightly surpassing posterior margin of T11 (right) or T9 (left) when folded backwards, basal 2 articles enlarged, less pilose; posterior 30 articles visibly longer than broad, ultimate article approx. 1.3 times longer than penultimate one (Fig. 3b). **Forcipules:** coxosternite subpentagonal (Fig. 4a), shoulders almost absent (steep), lateral margins straight; anterior margin set off as a rim by furrow; coxosternal teeth 8+8, median diastema well-developed, V-shaped, steep and narrow, porodont arising from a pit below the dental rim, situated lateral to the lateralmost tooth; base of porodont thinner then adjacent tooth, coxosternite sparsely setose anteriorly; setae moderately large, irregularly dispersed (Fig. 4b). Forcipular trochanteroprefemur, femur and tibia and proximal part of forcipular tarsungulum with several setae. Distal part of forcipular tarsungulum about 3 times longer than proximal part (Fig. 4a).

**Tergites**: T1 wider than long, subtrapeziform, wider anteriorly, posterior margin straight or slightly emarginated, marginal ridge with a small median thickening; TT3 and 5 more elongated than T1, posterior margin slightly emarginated medially, posterior angles rounded; posterior angles of T4 rounded; posterior margin of T8 slightly emarginated medially, angles rounded; TT6 and 7 with posterior angles abruptly rounded (Fig. 5a); TT9, 11, 13 with well-developed posterior triangular projections (Fig. 5b); posterior margin of TT10, 12, 14 slightly emarginated, posterior-most part densely setose; intermediate tergite subpentagonal, posterior margin deeply emarginated, lateral edges bent upwards, covered with setae; middle part of posterior third of tergite densely covered with setae; laterally, on both sides of the central setose area there are two specific seta-free regions (Fig. 6a, ***sfa***). All tergites smooth, setae present only on their lateral margins.

**Legs**: leg 15 longest; leg 14 approx. 25% longer than legs 1-12, leg 13 only slightly longer than legs 1-12; pretarsus of legs 1–14 with expanded fundus, larger posterior accessory claw (*approx.* 1/3^rd^ of fundus) and a slightly thinner and shorter anterior accessory claw (= spine, sensu Bonato et al. 2010) (Fig. 6b); pectinal (seriate) setae missing on tarsi 1 and 2 of leg 15, present in one short row on tarsus 2 of leg 14, and in one row on tarsus 1 and two rows on tarsus 2 of legs 1-13 (Fig. 7a); pretarsus of leg 15 without accessory spines (Fig. 7b). Length of podomeres of leg 15: coxa 1.5 mm, prefemur 3.7 mm, femur 4.0 mm, tibia 5.2 mm, tarsus 1 5.0 mm, tarsus 2 3.0 mm, pretarsus 0.25 mm. Prefemur of leg 15 with a large apically rounded proximal knob (Fig. 8) protruding mediad, latter slightly bent dorsad and bearing a peculiar cluster of long setae on tip (Fig. 9a); posterior edge with well defined circular protuberance at mid-distance between spines *a* and *p* dorsally, covered with long setae (Fig. 9b), rest of prefemur covered with sparse setae. Dorsal spine *p* on prefemur (but also in other podomeres and other legs) with characteristic bi- and tripartite tip (Fig. 10a). Legs 1-14 without particular modifications. **Coxal pores:** generally round, arranged in 4-5 irregular rows, pores of inner rows largest, size decreasing outwards; pores separated from each other by a distance more than, or equal to their own diameter; number of pores on leg-pair 12: L-36/R-33, 13 L-41/R-44, 14 L-52/R-49, 15: L-39/R-34 (Fig. 10b).

**Sternites:** all sternites smooth, subtrapeziform, with few sparse setae, mainly at lateral margins; posterior margins straight.

**Figure 11.**
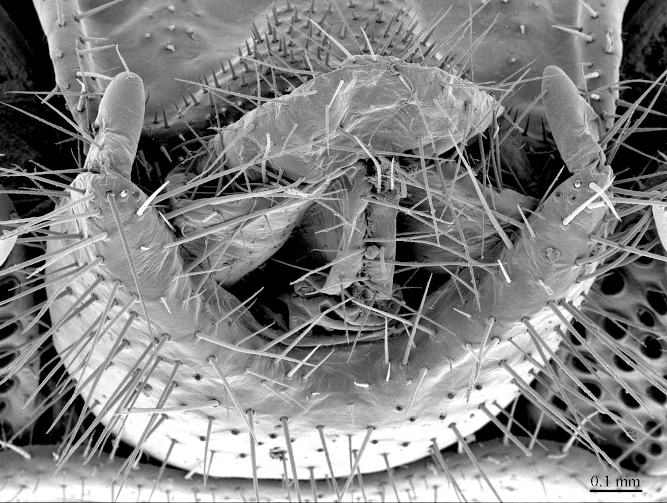
*Eupolybothrus cavernicolus* Komerički & Stoev sp. n., male paratype. Genitalia, posterio-dorsal view.

**Genitalia:** posterior margin of male first genital sternite deeply concave, up to half of its length, posterior margin densely covered with long setae, the rest of sternite sparsely covered with shorter setae; gonopod small, hidden behind the edge of first genital sternite, with 4-5 short setae (Fig. 11).

**Plectrotaxy:** as in Table 1.

##### Description of male paratypes

All characters like in the holotype, except the following: length of leg 15: prefemur 2.5 mm; femur 3.5 mm; tibia 4 mm; tarsus 1 3.7 mm; tarsus 2 2.5 mm; pretarsus 0.3 mm; ocelli: 1+12-1+13; antennae composed of 68-70 articles; coxosternal teeth: 6+7.

##### Description of adult female paratype

**Body length:** approx. 31 mm; leg 15 approx. 20-21 mm, or 68% length of body. **Color:** uniformly yellow-brownish to chestnut, head and T1 darker, legs yellowish, margins of tergites darker; distal parts of tarsungulum, coxosternal teeth and pretarsi of all legs dark brown to blackish.

**Head:** cephalic plate broader than long (3.9 x 3.5 mm, respectively), as wide as anterior part of T1; surface smooth, with several pits scattered throughout the head and giving rise to trichoid setae. Cephalic median sulcus contributing to biconvex anterior margin, marginal ridge with a median thickening; posterior margin slightly concave; transverse suture situated at about 1/3 of anterior edge; posterior limbs of transverse suture visible, connecting basal antennal article with anterior part of ocellar area. **Ocelli:** 18 blackish, subequal in size, in 3-4 rows. **Tömösváry’s organ:** moderately large (as large as or slightly larger than a medium ocellus), oval, situated slightly above the cephalic edge below the inferiormost row of ocelli. **Clypeus**: with a cluster of about 25 trichoid setae situated on the apex. **Antennae:** approx. 22 mm long, composed of 67 articles, reaching the middle of T10 when folded backwards, basal 2 articles enlarged, less setose; posterior 30 articles visibly longer than broad, ultimate article *approx.* 1.3 times longer than penultimate one. **Forcipules:** coxosternite subpentagonal, shoulders almost absent, lateral margins straight; anterior margin set off as a rim by furrow; coxosternal teeth 7+7, median diastema well-developed, V-shaped, subparallel and narrow, porodont arising from a pit below the dental rim, situated lateral to the lateralmost tooth; base of porodont thinner then adjacent tooth, coxosternite sparsely setose anteriorly; setae moderately large, irregularly dispersed. Medial side of forcipular trochanteroprefemur, femur and tibia and proximal part of forcipular tarsungulum setose. Distal part of forcipular tarsungulum about 3 times longer than proximal part.

**Tergites**: T1 wider than long, subtrapeziform wider anteriorly, posterior margin slightly concave; TT3 and 5 more elongated than T1, posterior margin slightly concave medially, posterior angles rounded; T2 almost entirely covered by T1, only posteriormost part surpassing the margin of T1; posterior margin of TT4 and 6 straight, posterior angles abruptly rounded; T7 rectangular, posterior margin straight, posterior angles abruptly rounded; T8 approx. 1.4 times longer than T7, posterior margin of T8 slightly concave medially, angles abruptly rounded; TT9, 11, 13 with a well-developed posterior triangular projections; TT10 and 12 subequal in size, approx. 1.2 times longer than T8, posterior margin slightly emarginated; posterior margin of T14 slightly emarginated, surface smooth, posterior-most part covered with just a few trichoid setae (much more setose in male, see Fig. 6a); intermediate tergite subpentagonal, posterior margin deeply emarginated, surface smooth, lateral edges bent upwards, a few trichoid setae emerging from the posterior and lateral edges; areas covered with spines and setae, as well as the specific setose free areas present in male (Fig. 6a, *sfa*) absent.

**Legs**: leg 15 longest, leg 14 latter approx. 25% longer than legs 1-12, leg 13 only slightly longer than legs 1-12; pretarsus of legs 1–14 with a more expanded fundus, larger posterior accessory claw (approx. 1/3^rd^ of fundus) and a slightly thinner and shorter anterior accessory claw (= spine, sensu Bonato et al. 2010); pectinal (seriate) setae missing on tarsi 1 and 2 of leg 15, present in one short row on tarsus 2 of leg 14, and in one row on tarsus 1 and two rows on tarsus 2 of legs 1-13; pretarsus of leg 15 without accessory spines. Leg 15 slender and elongate, without particular modifications. Bifurcated spines present irregularly on most podomeres. **Coxal pores:** generally round, forming 4-5 irregular rows, pores of inner rows largest, size decreasing outwards; pores separated from each other mostly by a distance more than or equal to their own diameter.

**Sternites:** subtrapeziform in shape, anterior part wider; lateral sides straight in all but ultimate sternite, where they are slightly convex; sternite surface smooth, shining, covered with a few sparse setae, mainly at lateral margins.

**Female gonopods:** densely setose, with 2+2 long and pointed spurs slightly bent outwards, and a single blunt claw; outer spur 1.4-1.5 times longer than the inner one, approx. 4 times longer than broad at base; 3-4 dorsal setae on article 1; 12 on article 2.

**Plectrotaxy:** as in Table 2.

##### Description of other female paratypes

Length: 19-22 mm; ocelli: 1+10–1+11; antennae composed of 65-68 articles; coxosternal teeth: 7+7. Tergites: TT8, 10 and 11 slightly emarginated; posterior margin of TT2, 4, 6, 7 straight. Legs: seriate setae missing on the tarsi 1 and 2 of leg 15, present in one short row only on posterior part of tarsus 2. Female gonopods: with 2+2 elongated sharply pointed spurs slightly bent outwards and a single blunt claw; 3-4 dorsal setae on article 1; 8 on article 2. Sometimes, a small, pointed spine occurs posteriorly in the middle of the first genital segment; so far, it has been detected only in two adult females [Kaczmarek (1973) reported similar spur in *Polybothrus ochraceus* (Folkmanova, 1936) (= *Eupolybothrus transsylvanicus*, cf. Stoev 2002)].

#### Diagnosis

The species can be readily distinguished from all other congeners by the following set of molecular and morphological characters: interspecific genetic distance in COI from the closest neighbour, *Eupolybothrus leostygis*: 14.5-15.4%; antennae moderately long (approx. 70% body length), comprised of 67-71 articles; 11-15 ocelli; 6+6-8+8 coxosternal teeth; tergites 9, 11, 13 with posterior triangular projections; intermediate tergite subpentagonal, posterior margin deeply emarginated, middle part of posterior third of tergite densely covered with setae; laterally, on both sides of the central setose area, there are two specific seta-free regions; pretarsus 15 without accessory spines; leg 15 long (approx. 70-75% body length), prefemur of male leg 15 with a large, apically rounded proximal knob protruding mediad, latter slightly bent dorsad and bearing a cluster of long setae on tip; distal part of prefemur with a well-defined circular protuberance covered with setae; posterior margin of male first genital sternite deeply emarginated, nearly as deep as half of the sternite’s length.

#### Description of the type locality

**Figure 12.**
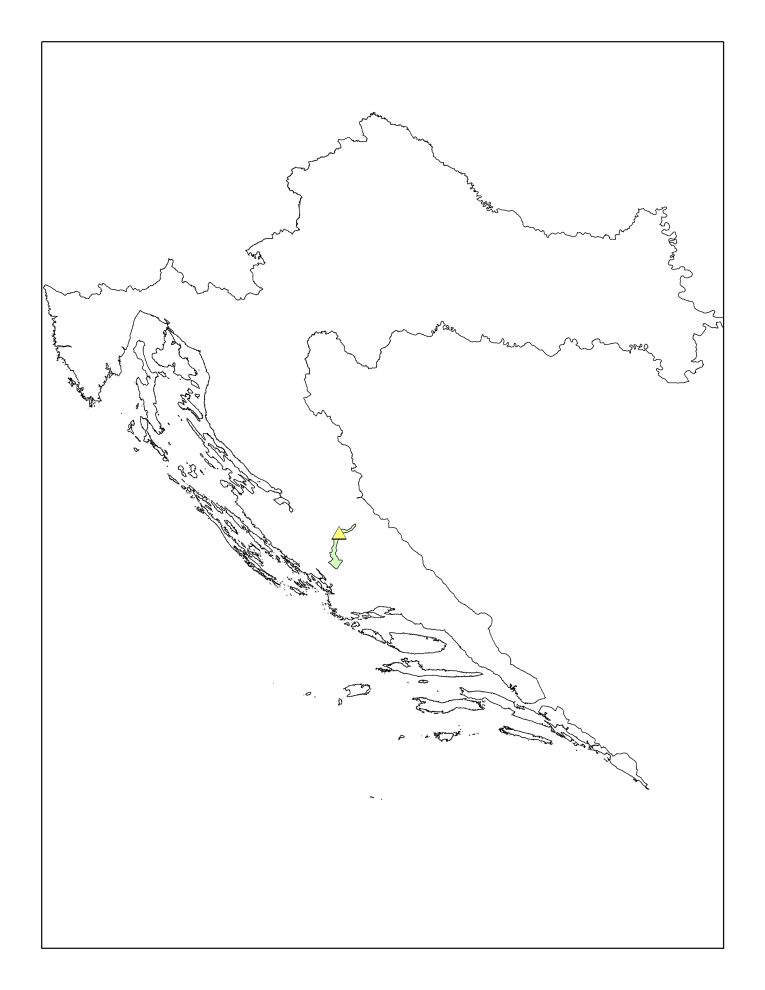
Map of Croatia showing the locality of *Eupolybothrus cavernicolus* Komerički & Stoev sp. n.

**Figure 13.**
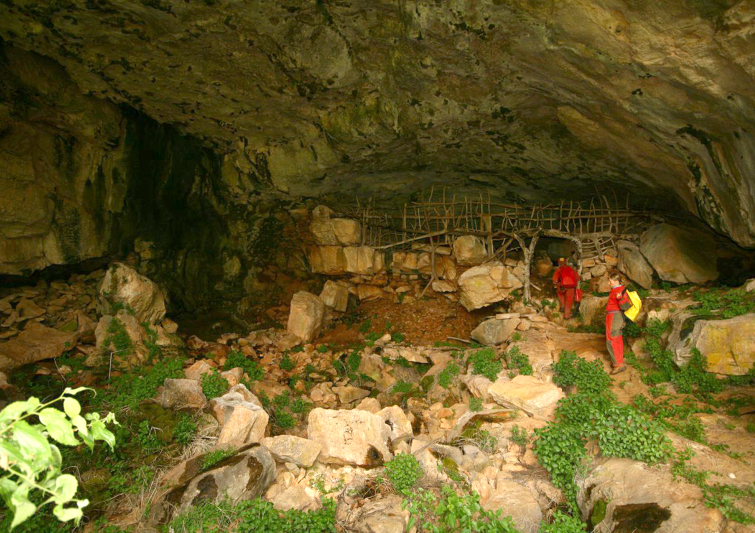
Entrance of cave Miljacka II, type locality of *Eupolybothrus cavernicolus* Komerički & Stoev sp. n.

*Eupolybothrus cavernicolus* Komerički & Stoev sp. n. is so far known only from the caves Miljacka II and Miljacka IV (= Špilja kod mlina na Miljacki), situated near the village of Kistanje, Krka National Park, Knin District, Croatia (Fig. 12). The two caves are situated close to each other and are formed in Middle Eocene to Early Oligocene conglomerate and marbly limestone. Miljacka II is the longest cave in the Krka National Park, with a large, spacious entrance and a total length of over 2800 m (Fig. 13). Most of the cave passages are under water except for approx. 300 m of main passage. From a hydrogeological point of view, cave Miljacka II contains a periodical spring, while cave Miljacka IV has a permanent water flow. The cave Miljacka IV has two entrances, one dry and one underwater, and a length of approximately 43 m. The land entrance is walled in and with a small door while inside the cave there is a thick drywall separating it in two parts. The climatic conditions in Miljacka II as measured on 4^th^ May 2010 and 8^th^ October 2010 are as follows: Tair = 12.5-13.7°C (Kestrel); RH = 100%; Tsediment = 12.5-13.2°C; Twater = 12.6-13.2°C; in Miljacka IV (measured on 4 May 2010): Tair = 13.1-13.6°C (Kestrel); RH = 100%; Tsediment = 12.5°C; Twater = 12.5°C. In Miljacka II, the specimens were collected in the aphotic zone, *approx.* 50 m from the cave entrance, in a passage where water never occurs in a periodic flow. In Miljacka IV, they were found closer to the entrance, under stones.

**Associated fauna:** Gastropoda: *Oxychilus cellarius* (O.F. Müller, 1774), *Hauffenia jadertina* Kuščer, 1933, *Hadziella sketi* Bole, 1961; Araneae: *Episinus cavernicola* (Kulczynski, 1897), *Nesticus eremita* Simon, 1879, *Tegenaria domestica* (Clerck, 1757), *Metellina merianae* (Scopoli, 1763), *Histopona* sp.; Pseudoscorpiones: *Chthonius tetrachelatus* (Preyssler, 1790), *Chthonius litoralis* Hadži, 1933, *Neobisium carsicum* Hadži, 1933, *Pselaphochernes litoralis* Beier, 1956; Opiliones: *Nelima troglodytes* Roewer, 1910; Acari: *Parasitus* sp.; Isopoda: *Monolistra pretneri* Sket, 1964, *Sphaeromides virei mediodalmatina* Sket, 1964, *Alpioniscus balthasari* (Frankenberger, 1937), *Cyphopleon kratochvili* (Frankenberger, 1939); Amphipoda: *Niphargus* sp.; Decapoda: *Troglocaris* sp.; Chilopoda: *Eupolybothrus tridentinus*, *Harpolithobius* sp., *Lithobius* sp., *Cryptops* sp.; Diplopoda: *Brachydesmus subterraneus* Heller, 1858; Collembola: *Troglopedetes pallidus* Absolon, 1907, *Heteromurus nitidus* (Templeton, 1835), *Pseudosinella heteromurina* (Stach, 1929), *Lepidocyrtus* sp.; Diplura: Plusiocampa (Stygiocampa) dalmatica Conde, 1959, Japygidae gen. spp.; Coleoptera: *Laemostenus cavicola mülleri* (Schaum, 1860), *Atheta spelaea* (Erichson, 1839); Orthoptera: *Dolichopoda araneiformis* (Burmeister, 1838), *Troglophilus ovuliformis* Karny, 1907, *Gryllomorpha dalmatina* Ocskay, 1832; Psocoptera: *Psyllipsocus* sp.; Lepidoptera: *Apopestes spectrum* (Esper, 1787); Amphibia: *Proteus anguinus* Laurenti, 1768; Chiroptera: a colony of bats, *Myotis capaccinii* (Bonaparte, 1837) (Marguš et al. 2012).

#### Etymology

*Cavernicolus* means “living in caves or caverns”, to emphasise that the species inhabits caves.

### Eupolybothrus leostygis

(Verhoeff, 1899)

http://dx.doi.org/10.5883/DS-EUPCAV

http://plazi.org:8080/GgSRS/html?CCEB9C62C87766E980DD858BC13468C8

KF715047

KF715051

KF715053

KF715060

KF715055

KF715056

KF715057

KF715058

999021822&tsn=true

Lithobius (Polybothrus) leostygis Verhoeff, 1899 - Verhoeff 1899: 451-452.

#### Other material

- **Occurrence:** recordedBy: J. Bedek; individualCount: 1; sex: male; lifeStage: adult; **Location:** country: Croatia; stateProvince: Dubrovnik-Neretva; locality: Jama u Zabirađu, Zabirađe, Osojnik; **Event:** samplingProtocol: hand collected; eventDate: 30 March 2008; **Record Level:** institutionCode: ZMUC; collectionCode: zmuc00029441

#### Notes

**Figure 16.**
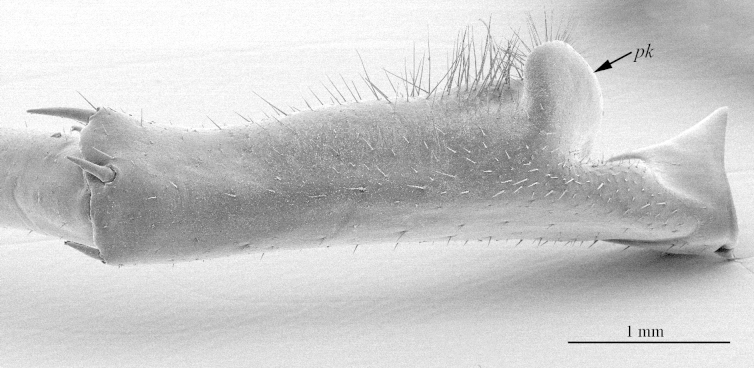
*Eupolybothrus leostygis* (Verhoeff, 1899), male: prefemur 15 showing the bare knob, dorsal view.

As the morphology of *Eupolybothrus leostygis* is still insufficiently known, we provide here scanning electron microscope images (Figs 14, 15, 16) based on an adult male specimen collected in Jama u Zabiradu Cave.

## Identification Keys

### Identification key to the species of Eupolybothrus (Schizopolybothrus) based on adult males

**Table:** 

1	Six poorly defined, feebly pigmented ocelli [original description]	*Eupolybothrus leostygis*
–	10-25 pigmented ocelli	2
2	Leg 15 with a large knob on prefemur (Figs 8b, 16)	3
–	Leg 15 without such knob (Fig. 17a) [original description]	*Eupolybothrus tabularum*
3	Prefemoral knob apically incised forming two rounded and densely setose processes (Fig. 17b) [original description]	*Eupolybothrus excellens*
–	Prefemoral knob simple (Fig. 16)	4
4	Prefemoral knob with a cluster of setae (Fig. 8a)	*Eupolybothrus cavernicolus* sp. n.
–	Prefemoral knob without such cluster of setae (Fig. 16)	5
5	Antennae with 50-60 antennal articles	6
–	Antennae with 70-83 antennal articles	7
6	Prefemoral knob poorly developed (Fig. 18a) [original description]	*Eupolybothrus caesar*
–	Prefemoral knob large (Fig. 18b) [original description]	*Eupolybothrus spiniger*
7	Coxosternal teeth: 10+10-11+11; 1 ventral spine on tibia of leg 15 [original description]	*Eupolybothrus stygis*
–	Coxosternal teeth: 8+8-9+9; 2 ventral spines on the tibia of leg 15	8
8	Antennae with 74 antennal articles, ocelli 1+14 [original description]	*Eupolybothrus acherontis*
–	Antennae with more than 81-83 articles, ocelli 1+18-1+19 [original description]	*Eupolybothrus acherontis wardaranus*

## Analysis

### Molecular delimitations

**Figure 19.**
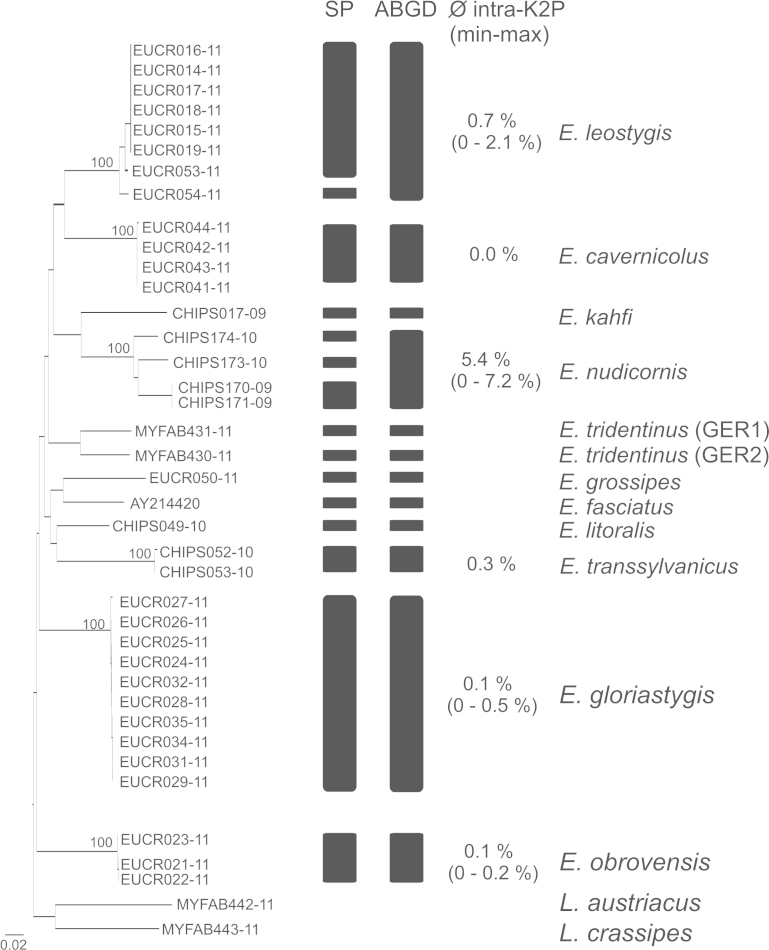
Delineation of *Eupolybothrus* species – Neighbor joining tree K2P distances. Visualised are the clusters obtained from the reversed Statistical Parsimony (SP) method and the Automatic Barcoding Gap Discovery (ABGD) procedure. Bootstrap support for the identified lineages are given above. The intraspecific genetic variability is given for each cluster. Source data is available in Suppl. material 1.

The ABGD approach clustered the 37 *Eupolybothrus* specimens into 12 groups (Fig. 19). Ten of them agreed with morphospecies designations: *Eupolybothrus grossipes*, *Eupolybothrus leostygis*, *Eupolybothrus kahfi*, *Eupolybothrus litoralis*, *Eupolybothrus nudicornis*, *Eupolybothrus fasciatus*, *Eupolybothrus transsylvanicus*, *Eupolybothrus obrovensis*, *Eupolybothrus gloriastygis* and *Eupolybothrus cavernicolus* Komerički & Stoev sp. n. The remaining two genetic clusters are each formed by a specimen of the morphospecies *Eupolybothrus tridentinus* from Germany. The reversed SP networks support most of the ABGD results and morphospecies assignments, but split both *Eupolybothrus leostygis* and *Eupolybothrus tridentinus* in two and *Eupolybothrus nudicornis* in three clusters, respectively. We follow a conservative approach here and refer to the ABGD results, which largely correspond to morphology, *Eupolybothrus tridentinus* being the only exception which could suggest a case of cryptic diversity and will require further investigation. All delineated groups have a bootstrap support of 100 in the NJ-tree topology. The interspecific K2P genetic distance ranges from 10.7 % for the species pair *Eupolybothrus tridentinus* (GER1) – *Eupolybothrus tridentinus* (GER2) to 24.5 % for *Eupolybothrus grossipes* – *Eupolybothrus cavernicolus* Komerički & Stoev sp. n. (Table 3). Intraspecific K2P genetic distance is maximal for *Eupolybothrus nudicornis* (7.2 %) and minimal for individuals of *Eupolybothrus obrovensis* and *Eupolybothrus gloriastygis* and the species *Eupolybothrus cavernicolus* Komerički & Stoev sp. n. (0.0 %) (Fig. 19).

### Transcriptome analysis and annotation

The raw data was first filtered by removing inadequate reads with: 1) adapter contamination; 2) ≥10 Ns; 3) ≥50 base pairs of low quality (quality value <65). The resulting 2 Gb of clean data were processed into subsequent assemblies using SOAPdenovo_trans (Xie et al. 2013) under default parameters. The abundance information was provided directly by SOAPdenovo_trans, and played no roles in the subsequent analysis steps. A total of 67,785 scaffolds were produced with an average length of 812 bp and N50 of 1,448 bp [see GigaDB (Stoev et al. 2013)]. Subsequent annotation was conducted by tracing homologs against currently available databases, including Nr, SwissProt and COG. Using this method, 22,866 scaffolds were functionally annotated (Fig. 20a, b, c). Annotated genes were then translated to peptide sequences via CDS prediction according to their blast results using GeneWise (Birney et al. 2004) (see GigaDB (Stoev et al. 2013)). Using orthoDB (http://cegg.unige.ch/orthodb6) (Waterhouse et al. 2012), 2,188 one to one orthologs were filtered out from four selected arthropod genomes: *Drosophila melanogaster* Meigen, 1830, *Daphnia pulex* (Linnaeus, 1758), *Ixodes scapularis* Say, 1821 and *Strigamia maritima* (Leach, 1817). HaMstR (Ebersberger et al. 2009) was applied to search corresponding orthologous genes in our transcriptome data, delivering 1,668 predicted orthologs of both nucleotide and protein sequences (see GigaDB (Stoev et al. 2013)).

## Discussion

### Taxonomic affinities

According to the division of the subgenera of *Eupolybothrus* of Jeekel (1967), *Eupolybothrus cavernicolus* Komerički & Stoev sp. n. falls into subgenus *Schizopolybothrus* Verhoeff, 1934, characterized by the presence of triangular projections on tergites 9, 11, 13, a VCm spine on leg 15, one or more VCa spines and a single claw on the pretarsus of leg 15. The same author further distinguishes three species groups in the subgenus based on the morphology of male gonopods and presence/absence of modifications on leg 15:

- Group I, characterized by short male gonopods and presence of a large knob on male prefemur 15, currently including *Eupolybothrus caesar* (Verhoeff, 1899), from Bosnia-Herzegovina, Albania, mainland Greece (incl. Ionian Is.) and Macedonia (FYROM); *Eupolybothrus spiniger* (Latzel, 1888), from Bosnia-Herzegovina; *Eupolybothrus acherontis* (Verhoeff, 1900), from Bosnia-Herzegovina (*Eupolybothrus acherontis acherontis*) and Macedonia (FYROM) (*Eupolybothrus acherontis wardaranus* (Verhoeff, 1937)); *Eupolybothrus stygis* (Folkmanova, 1940), from Bosnia-Herzegovina; and *Eupolybothrus leostygis*, from Croatia and Bosnia-Herzegovina (see Kos 1992, Stoev 1997, Zapparoli 2002). Here also belongs a new cave-dwelling species from Velebit, Croatia, recently discovered by AK and the CBSS team, whose description is currently in progress. While *Eupolybothrus caesar* and *Eupolybothrus leostygis* have recently been validated and re-described (see Eason 1983, Zapparoli 1984, Zapparoli 1994), the status of the other four taxa remains uncertain (see e.g., Stoev 2000, Stoev 2001, Stoev et al. 2010)
- Group II, lacking any specific modifications on male legs while gonopods are also short, encompassing *Eupolybothrus tabularum* (Verhoeff, 1937) from the Western Alps and *Eupolybothrus excellens* (Silvestri, 1894) from the Ligurian Apennines.
- Group III, characterized by the long gonopods and dorsal furrow on male prefemur 15, with *Eupolybothrus zeus* (Verhoeff, 1901) from Central Greece and *Eupolybothrus sissii* (Kanellis, 1959) from Euboea Island, Greece. Both species are currently considered junior synonyms of the widespread Carpathian-Balkan species Eupolybothrus (Mesobothrus) transsylvanicus (cf. Zapparoli 1994).

Jeekel’s division of the genus (Jeekel 1967) is quite artificial and does not reflect real evolutionary relationships as it is merely based on a few morphological traits. Some species were certainly misplaced in these groupings, as for example *Eupolybothrus excellens*, of which, males show noticeable modifications on leg 15 (see Fig. 17b). Two other species, *Eupolybothrus zeus* and *Eupolybothrus sissii*, were even excluded from *Schizopolybothrus* (cf. Zapparoli 1994, Zapparoli 2002). Showing a prominent prefemoral knob on male leg 15 and having relatively short gonopods, *Eupolybothrus cavernicolus* Komerički & Stoev sp. n. unquestionably belongs in Group I, as defined by Jeekel (1967). The new species can be readily distinguished from other members of Eupolybothrus (Schizopolybothrus) by the presence of a large proximal knob surmounted by a characteristic cluster of setae, and distal setose protuberance of male prefemur 15. In addition, the species presents a different arrangement of spiniform setae on the intermediate tergite.

### Micro-computed tomography and ‘cybertype’ notion

The new generation imaging technologies, such as magnetic resonance imaging (MRI) and micro-computed tomography (micro-CT) are opening new horizons in biology (Mietchen et al. 2008, Ziegler et al. 2008). Micro-CT is becoming widely used in comparative, developmental and functional biology (see e.g., Metscher 2009a, Metscher 2009b, Wojcieszek et al. 2012), paleontology (Błażejowski et al. 2011, Edgecombe et al. 2012), molecular biology (Metscher and Müller 2011) and taxonomy (Faulwetter et al. 2013, Michalik et al. 2013). By employing micro-CT scans in taxonomy, important morphological and anatomical characters can be examined in their natural position without damage to the original specimen. This allows researchers to re-assess character shape and functionality or even discover new diagnostic characters (Ziegler et al. 2010, Zimmermann et al. 2011, Faulwetter et al. 2013). To make type material continuously and simultaneously available to taxonomists and to improve access to high-quality morphological data, Faulwetter et al. (2013) suggested the creation of high-resolution virtual morphological and anatomical data libraries allowing reconstruction and interactive manipulation of type specimens, the so-called ‘cybertypes’.

The ‘cybertype’ notion is herewith tested for the first time with the newly described taxon (Fig. 21), for which a rich image library has been created to allow its subsequent recognition, virtual manipulation and reuse. This image library, from which the 3D model is created, has been deposited in the GigaScience database, GigaDB, as a zip and a gzipped tar archive containing BMP images (Stoev et al. 2013). The 3D model was converted into an AVI file, using the flight recorder of CTVox, and disseminated, along with the video of the living specimen (Fig. 22) through YouTube. According to Faulwetter et al. (2013), a ‘cybertype’ should be linked to the original type material and be retrievable and freely accessible. We comply with these requirements by a) including a set of Darwin Core files along with the deposited volumetric data which describe the attributes and deposition of the physical type material and b) using a CCZero license and rich metadata to make the "cybertype" retrievable and reusable. Furthermore, through the same set of Darwin Core files, the morphological data are also linked to the transcriptomic data at GigaDB, effectively extending the ‘cybertype’ concept and providing direct links to other data describing type material of the same species.

### Data management and release

Whereas a lack of reference genomes in non-model organisms has hampered genetic and phylogenomic studies, transcriptomes may present a time and cost-effective substitute to whole genome sequencing for these types of studies and an efficient way to produce massive amounts of gene sequence data. While transcriptomic studies of centipede species, e.g. *Alipes grandidieri* Lucas, 1864 (Chilopoda; Scolopendromorpha), exist in the literature (Riesgo et al. 2012), centipede genome data in public and accessible repositories are still scarce and difficult to find. To address this deficiency, and to produce a model of an accessible resource for the community, all of the transcriptomic data have been made available under the highest metadata standards, both in relevant community specific databases (raw data in the SRA [SRA project accession: ERP003841] and transcriptomic in ArrayExpress [accession E-MTAB-1859]), as well as GigaDB (Stoev et al. 2013). GigaDB collects together all of the genomic and morphological data, and utilises the large computing infrastructure of the BGI and Aspera data transfer capabilities, able to host and deliver much larger and heterogeneous datasets than other repositories (Sneddon et al. 2012). Datasets are also issued with DOIs, which are discoverable through the DataCite metadata search engine and Thomson Reuters Data Citation Index, and can be integrated into a publication or independently cited.

In addition to making data publicly available, it is crucial to provide rich metadata to enable data interoperability and reuse. As there is only one transcriptome available, it is not possible to include additional ‘factor’ information. However, by including sequence reads, experimental design, protocols and processed data we were able to produce the maximum amount of (4*) MINSEQE compliant metadata (Brazma 2009). To maximise its interoperability, the metadata are also available from GigaDB in ISA-TAB format (Sansone et al. 2012).

For volumetric data created by techniques such as micro-CT and micro-MRI, no community standards exist yet. The DICOM standard (Digital Imaging and Communications in Medicine, http://dicom.nema.org/) used by the medical community is not tailored for taxonomic purposes, thus its usefulness for this research field still has to be investigated (Faulwetter et al. 2013). However, even in the absence of widely accepted standards, we provide rich metadata for the micro-CT data, based on the metadata descriptors at Morphosource (http://morphosource.org). The same set of descriptors has been used by GigaDB, where we also applied the ISA-TAB format in order to ensure re-usability and interoperability of the data (Sansone et al. 2012), describing all parameters and settings used to create the data. The data package of micro-CT deposited at GigaDB thus contain:

- MicroCT image stack available in 2 different formats:
  - Several ZIP files, each contains 500 bmp images, the scanning log documentation file and Darwin Core type specimen data.
  - A single gzipped TAR archive of all 3876 bmp images, the scanning log documentation file and Darwin Core type specimen data.
- Documentation of the scanning and reconstruction process through ISA-TAB metadata provided by GigaDB and the inclusion of the scanning log file with the ‘cybertype’.
- Specimen data in Darwin Core format and link to the location of the physical material and the transcritomic data through Darwin Core comma-separated value format (CSV) files:
  - Eupolybothrus_cavernicolus_sequenced_vaucher_ paratype.csv
  - Eupolybothrus_cavernicolus_micro-CT_vaucher_ paratype.csv
  - Eupolybothrus_cavernicolus_all_types.csv
- ISA-TAB metadata that ensure retrievability and interoperability.

In combination with the Darwin Core files describing the specimen data, we thus fully annotate and document the ‘cybertype’ of *Eupolybothrus cavernicolus* Komerički & Stoev sp. n. The generation of large molecular and morphological data pools that originate from type specimens increases the applicability of taxonomic data in other scientific disciplines such as comparative morphology, evolutionary biology, medicine, ecology. The new holistic approach raises important questions and shows up new directions for developments of biodiversity data management about the lack of mechanisms for cross-linking molecular and morphological data and global metadata standards for micro-CT and transcriptomic data, as well as absence of reliable data repositories for micro-CT image libraries.

Also, as a pilot project, we annotate all currently valid Eupolybothrus (Schizopolybothrus) species with their original descriptions that were extracted from the original publications through applying optical character recognition (OCR) and additionally tagged by using Golden Gate software (Sautter et al. 2007). All species treatments are deposited at Plazi. This represents part of a more ambitious project aiming at digitization of all species descriptions and important taxonomic treatments of *Eupolybothrus* species that is currently being carried out in the framework of the pro-iBiosphere project (http://www.pro-ibiosphere.eu).

To create reliable links between the published sequence IDs and BOLD, an online dataset DS-EUPCAV was generated in the BOLD system, through which the respective Barcode Index Numbers (BINs) of the specimens barcoded for this study may be tracked (for the BIN concept see Ratnasingham and Hebert 2013). All COI sequences were registered in GenBank, following a newly launched metadata standard in the GenBank taxonomy database that flags sequences of type specimens.

### Conclusions

This study demonstrates a holistic approach to the description of a new taxon, extending the conventional written description and two-dimensional illustrations with an array of different information types. While this novel approach contributes to the different aspects of the species' identity, its main aim is to provide an integrated approach to handling and publishing large data sets associated with a taxon. The generation of large molecular and morphological data pools that originate from type specimens increases the applicability of taxonomic data in other scientific disciplines such as comparative morphology, evolutionary biology, medicine, ecology, and others.

The concept of a “cybertype” is discussed in the study, but at the same time new questions arise, pertaining to the definition of such a “cybertype” and they will have to be addressed by the taxonomy community. Several different kinds of data belonging to the “cybertype” concept are treated in this study, from free text to sequence data, and from images to volumetric data. Questions have to be addressed such as whether a cybertype should only be restricted to morphological data, what data can be used to constitute a cybertype and whether a cybertype can be composite (i.e. consisting of several data types) or even distributed (different parts of the data residing on different physical servers). Further problems to be addressed are the lack of appropriate mechanisms for cross-linking molecular and morphological data, as well as the absence of global metadata standards and reliable data repositories for micro-CT image libraries. The metadata descriptors for micro-CT files used by the Morphosource and GigaDB repositories are a good starting point for that, as is the use of ISA-TAB to integrate everything together. Whatever the answers to these questions, there is one mandatory requirement for data that we can already identify: discoverability and accessibility.

With complex taxon descriptions such as the present one, we are entering new dimensions of data volumes that have to be managed properly to realise their true value. The deposition of large data pools in appropriate repositories is not yet straightforward, and such initiatives have started to emerge only recently. It is our task to ensure from the beginning that they do not develop into isolated data worlds but that they support community standards, describing the datasets in a way that they can be retrieved and cross-linked. Currently, even in modern taxon descriptions, different pieces of data are only linked through a central locus: the published article. In a future, data-centric world of taxonomy, articles published through next generation journal workflows will become an even more important node in a linked network of data elements describing the taxon. These data elements have to be defined and made accessible through persistent identifiers – not unlike the traditional practice for physical specimens that are accessible through their museum accession number. In combination with rich metadata standards, taxonomy will thus open itself up to the semantic Web with its possibilities for intelligent, complex queries.

In this study, we have taken a first step towards that direction. All data have been deposited in publicly accessible repositories, such as GigaDB, NCBI, BOLD, Morphbank and Morphosource, and the respective open licenses used ensure their accessibility and re-usability. GigaDB in this example provides direct links between the genomic and micro-CT data, through a Darwin Core CSV dataset describing the type specimens, as well as capturing all of the metadata in the interoperable ISA-TAB format. Molecular data and images are annotated with rich metadata to ensure discoverability and reuse. Techniques such as micro-CT are, however, still in their infancy, and no standardised metadata schemas exist yet – a gap that needs urgently to be addressed by the community if we are to avoid a proliferation of isolated datasets.

Taxonomy is at a turning point in its history. New technologies allow for creation of new types of information at high speed and in gigantic volumes, but without clear rules for communication standards, we will not be able to exploit their full potential. We need to focus our efforts on linking these bits and pieces together, by documenting them, by standardising them and by making them retrievable. If such an infrastructure is in place, unforeseen analytical powers can be unleashed upon these data, creating a revolution in our abilities to understand and model the biosphere.

## Supplementary Material

XML Treatment for Eupolybothrus cavernicolus

XML Treatment for Eupolybothrus leostygis

## Figures and Tables

**Figure 2a.**
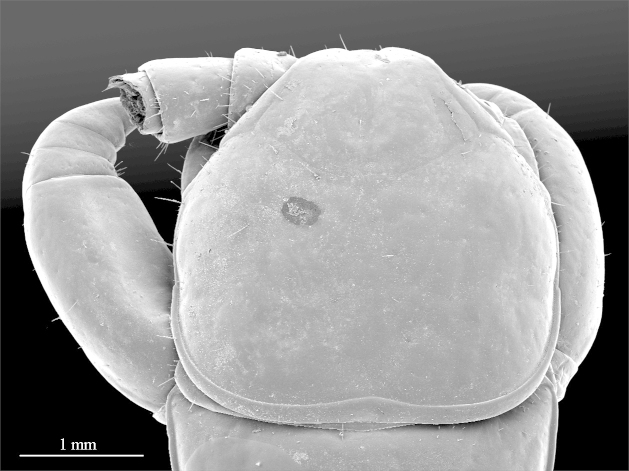
cephalic plate, dorsal view

**Figure 2b.**
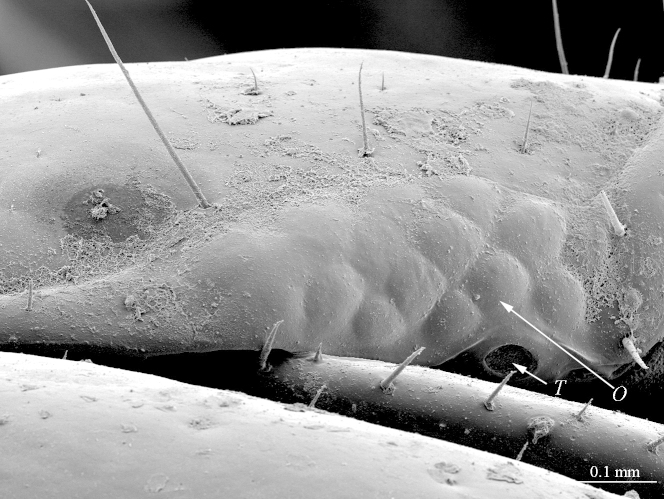
ocelli and Tömösváry’s organ. Abbreviations: ocellus (*O*) and Tömösváry’s organ (*T*)

**Figure 3a.**
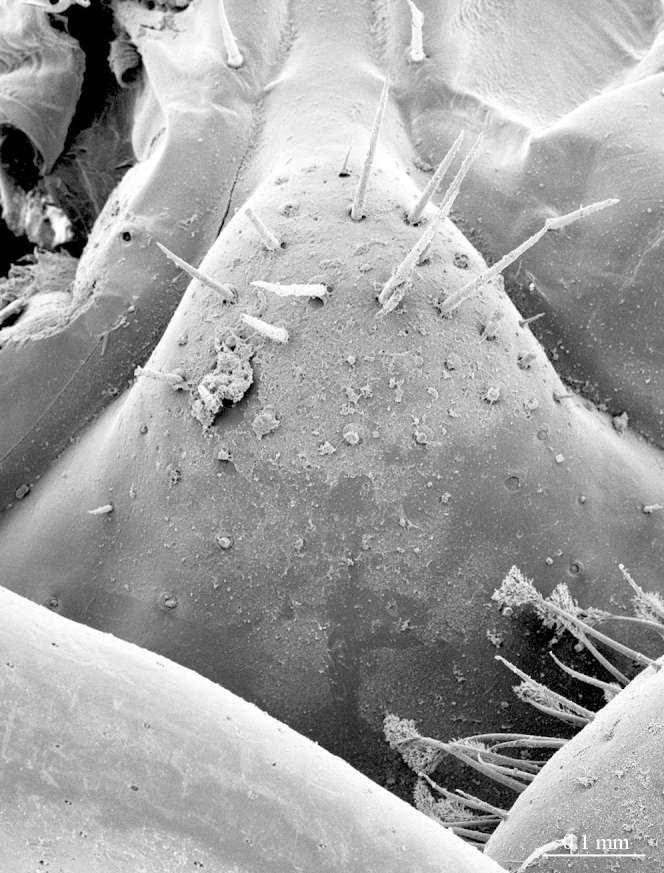
clypeus, ventral view; most setae broken off

**Figure 3b.**
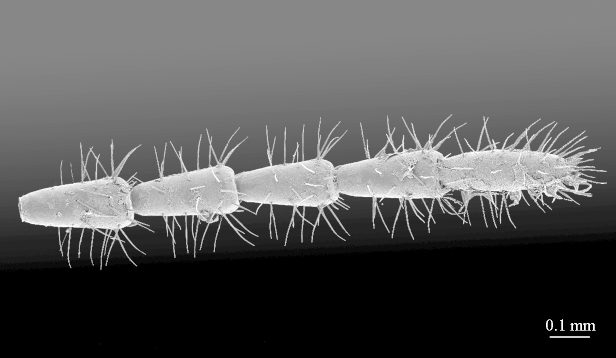
tip of antenna

**Figure 4a.**
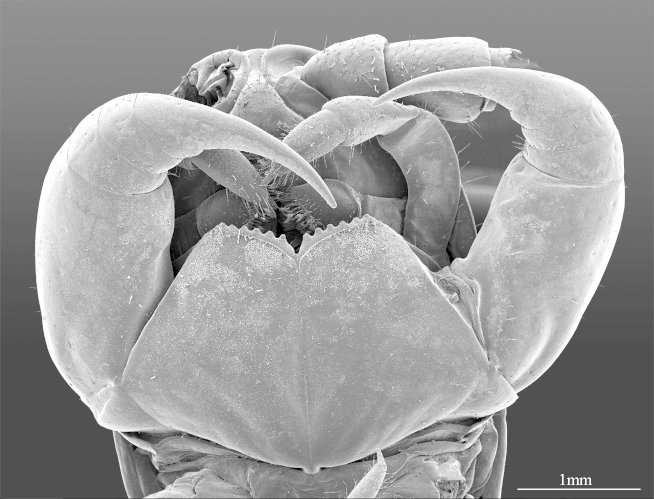
forcipules, ventral view

**Figure 4b.**
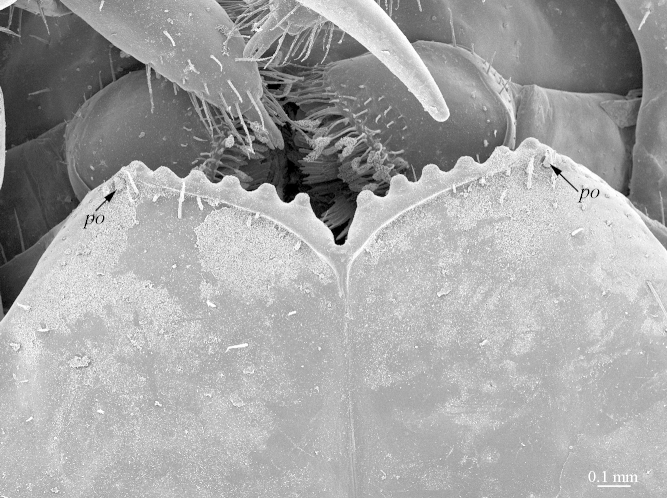
close up of coxosternum, ventral view. Abbreviations: porodonts (*po*).

**Figure 5a.**
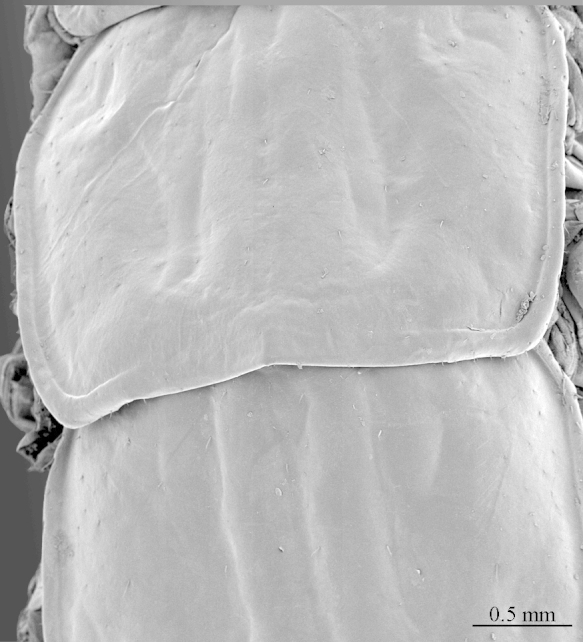
tergite 7, dorsal view

**Figure 5b.**
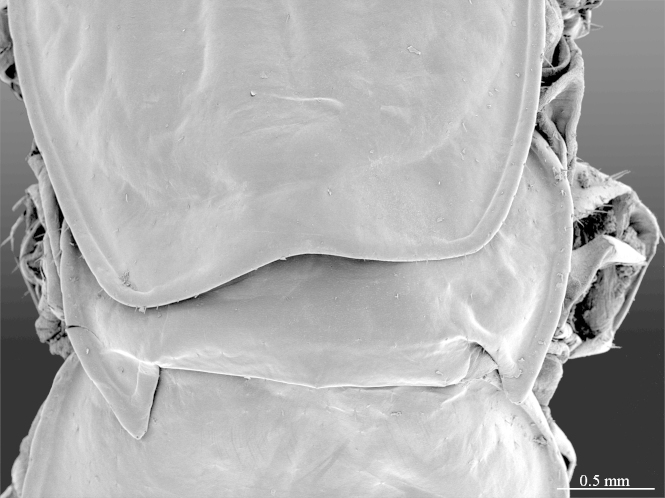
tergites 12-13, dorsal view

**Figure 6a.**
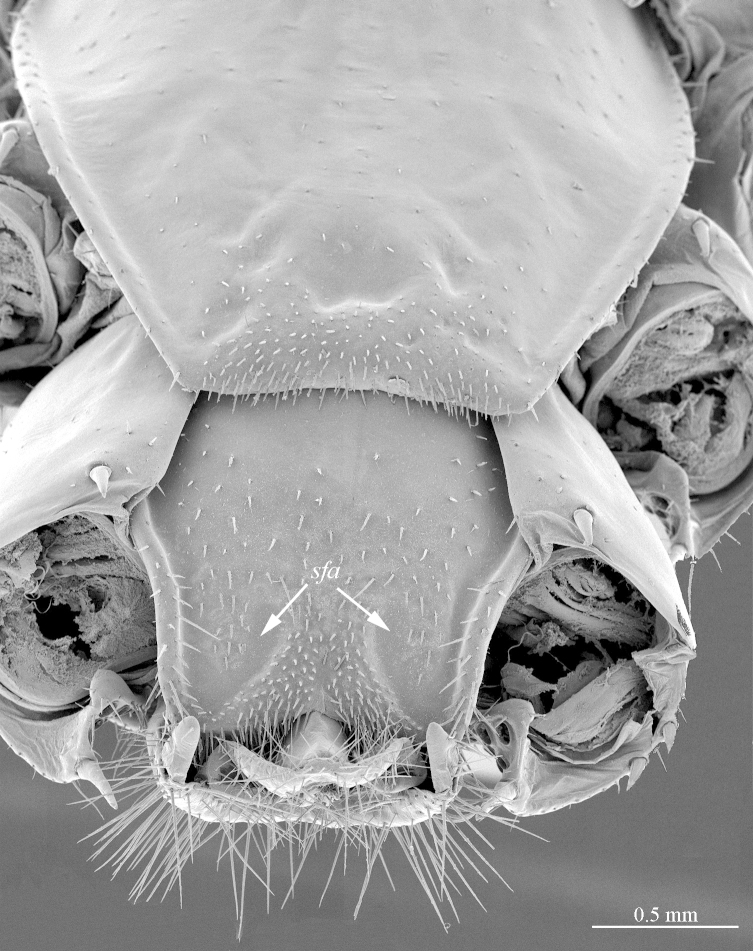
tergite 14 and intermediate tergite, posteriodorsal view. Abbreviations: seta-free areas (*sfa*).

**Figure 6b.**
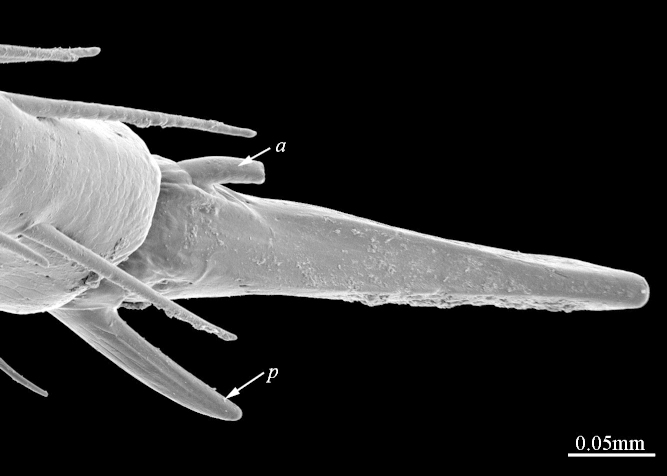
pretarsus of leg 10, ventral view. Abbreviations: anterior accessory claw (*a*), posterior accessory claw (*p*).

**Figure 7a.**
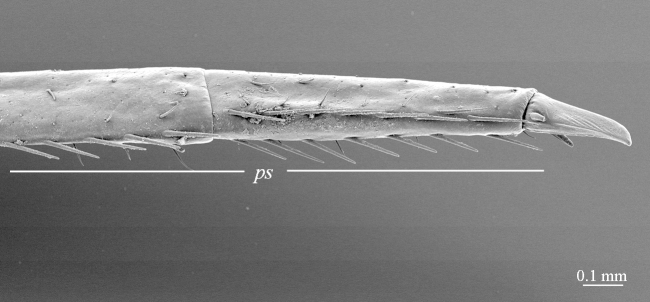
tarsus 1, tarsus 2 and pretarsus of leg 10, lateral view. Abbreviations: pectinal setae (*ps*).

**Figure 7b.**
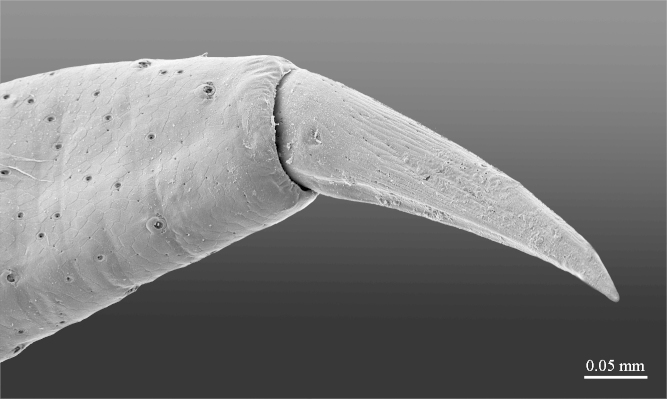
pretarsus of leg 15

**Figure 8a.**
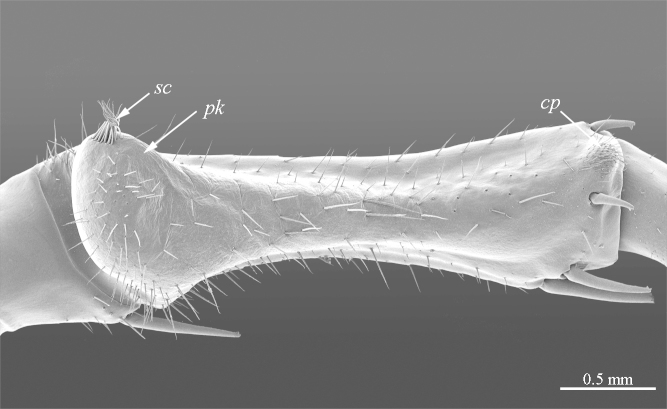
prefemur 15, mesoventral view. Abbreviations: prefemoral knob (*pk*), circular setose protuberance (*cp*), cluster of setae (*sc*).

**Figure 8b.**
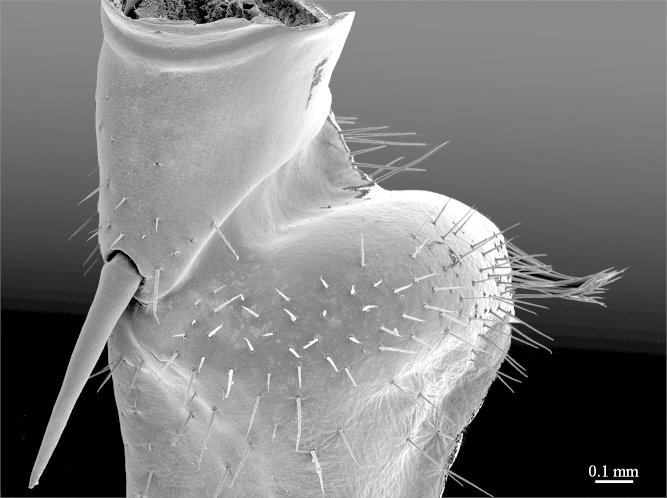
close up of the prefemoral knob, ventral view

**Figure 9a.**
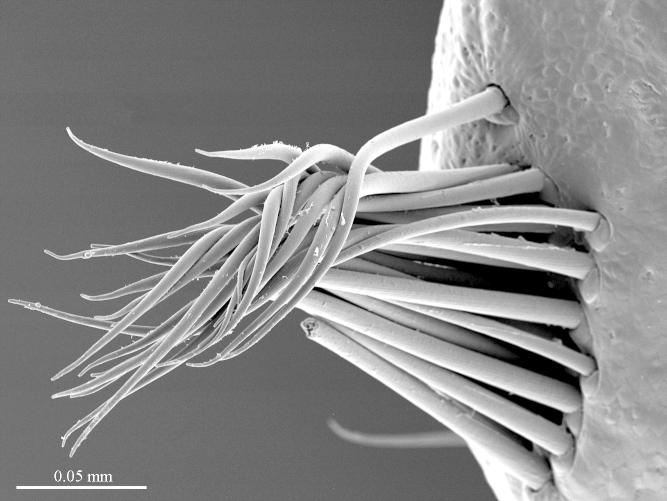
close up of the clusp of setae on male prefemur 15

**Figure 9b.**
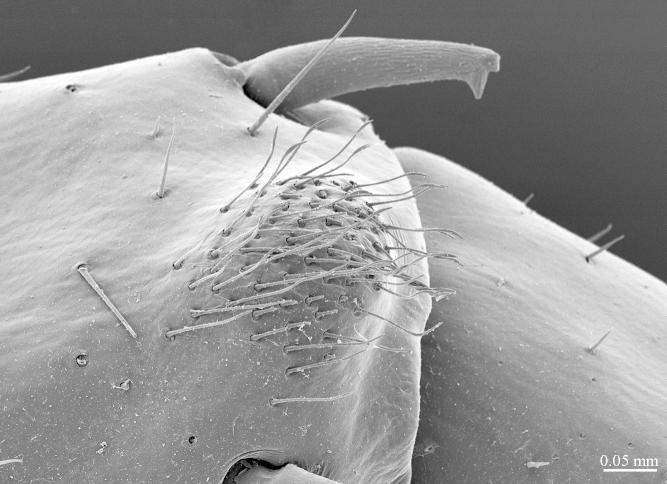
close up of the setose protuberance on male prefemur 15

**Figure 10a.**
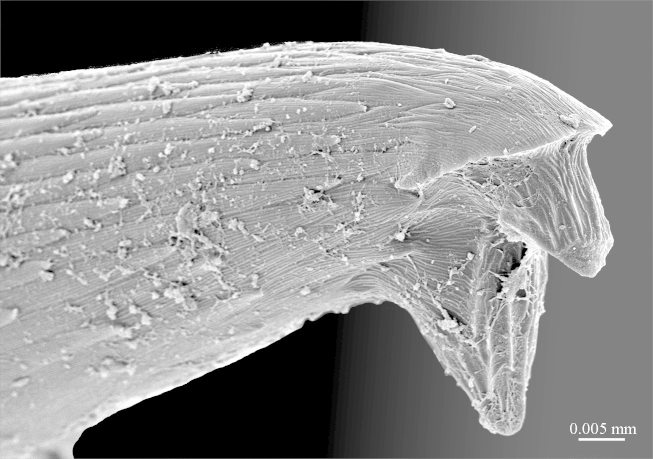
close up of the tip of prefemoral spine *p*

**Figure 10b.**
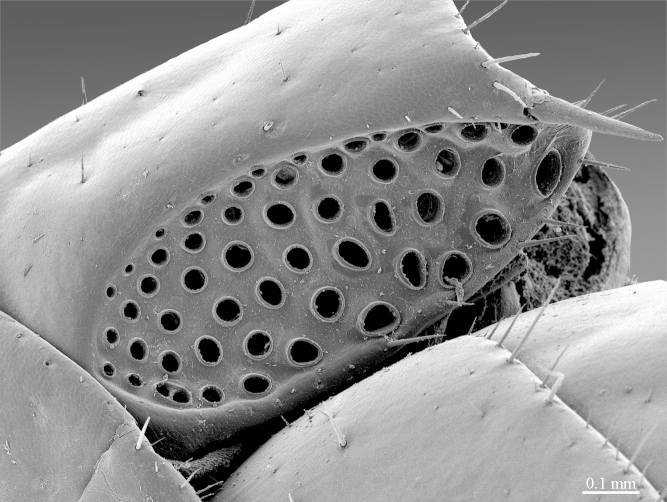
coxal pore pit, meso-ventral view

**Figure 14a.**
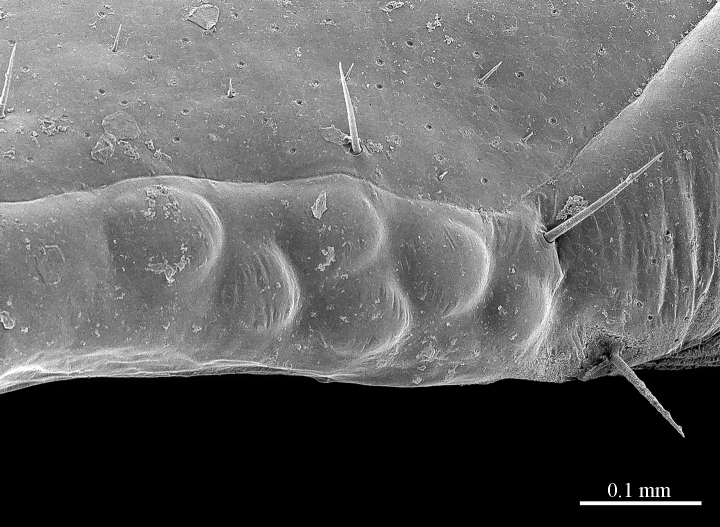
ocelli

**Figure 14b.**
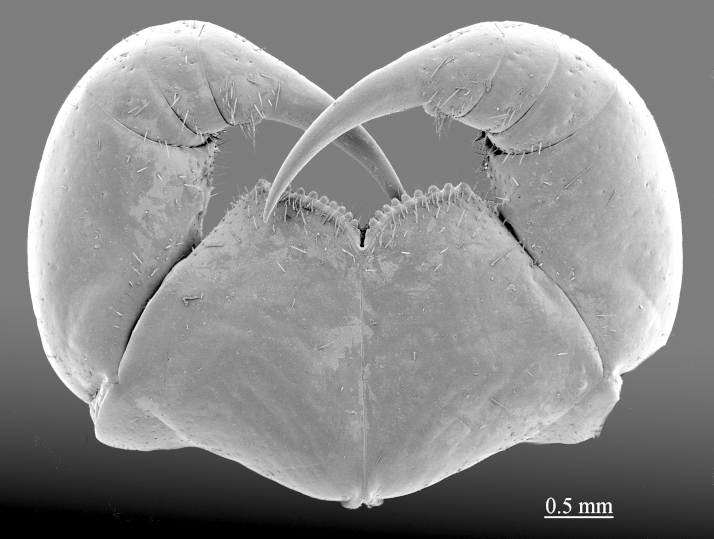
forcipules, ventral view

**Figure 15a.**
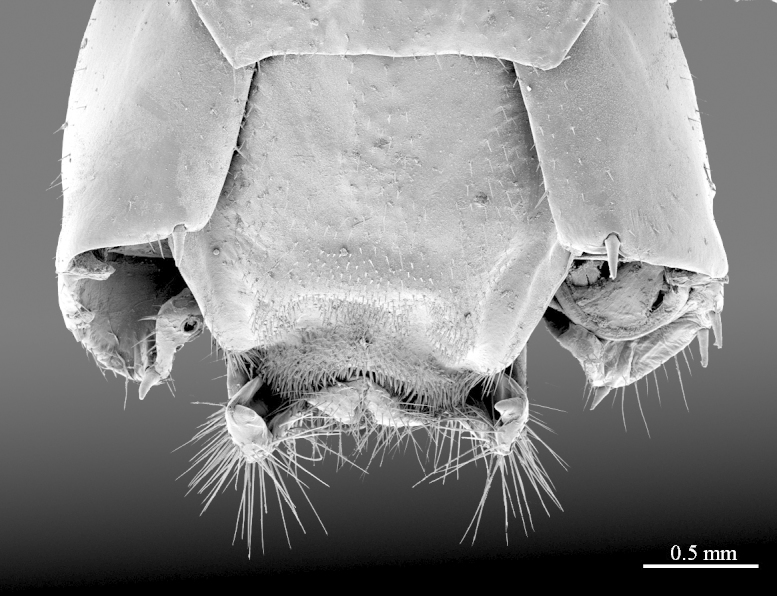
tergite 14 and intermediate tergite, dorsal view

**Figure 15b.**
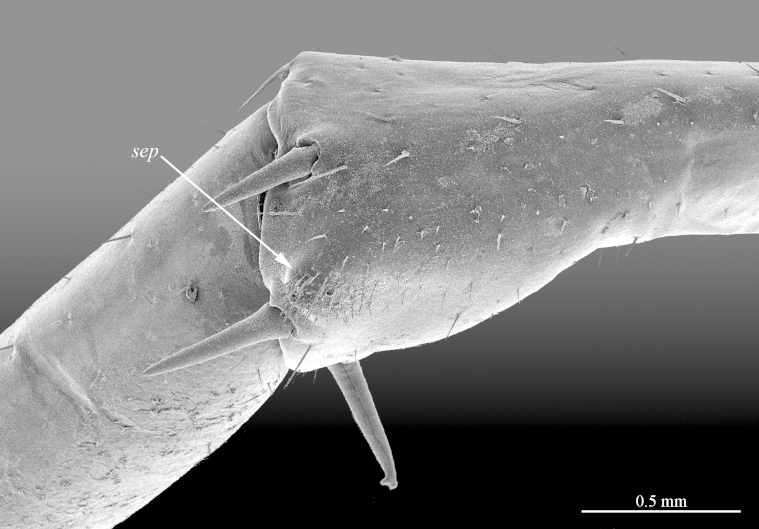
close up of posterior part of prefemur of leg 14 showing the expanded distal part bearing feebly defined setose protuberance

**Figure 17a.**
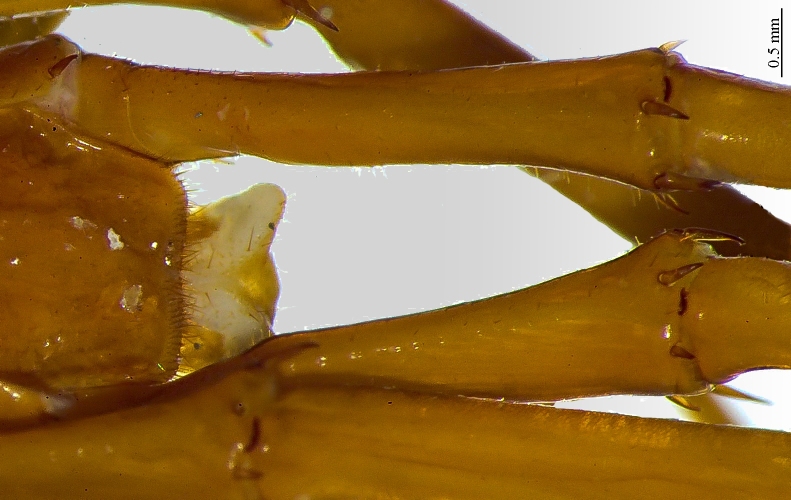
Eupolybothrus tabularum

**Figure 17b.**
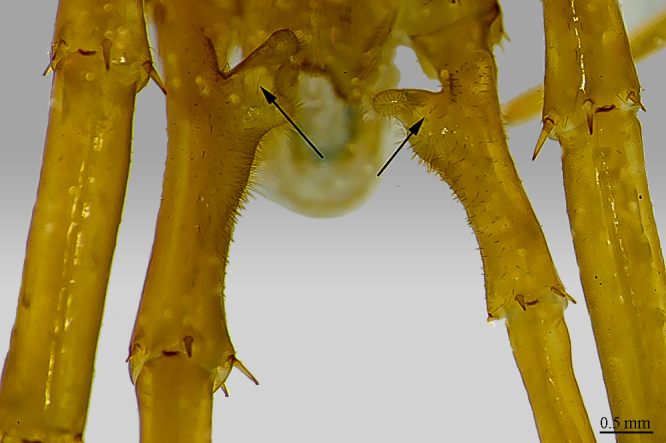
Eupolybothrus excellens

**Figure 18a.**
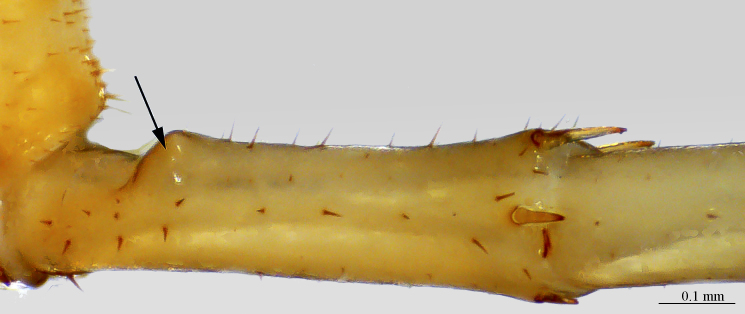
Eupolybothrus caesar

**Figure 18b.**
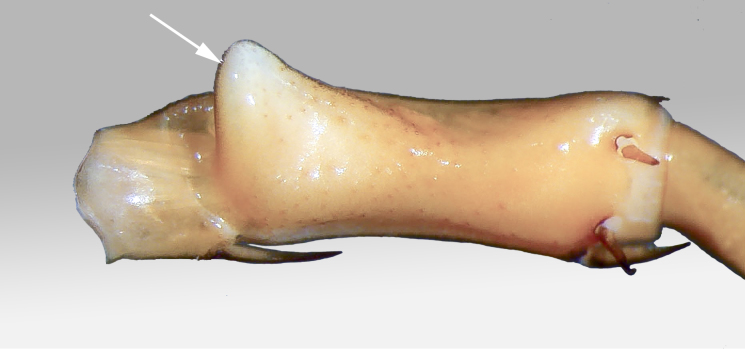
Eupolybothrus spiniger

**Figure 20a.**
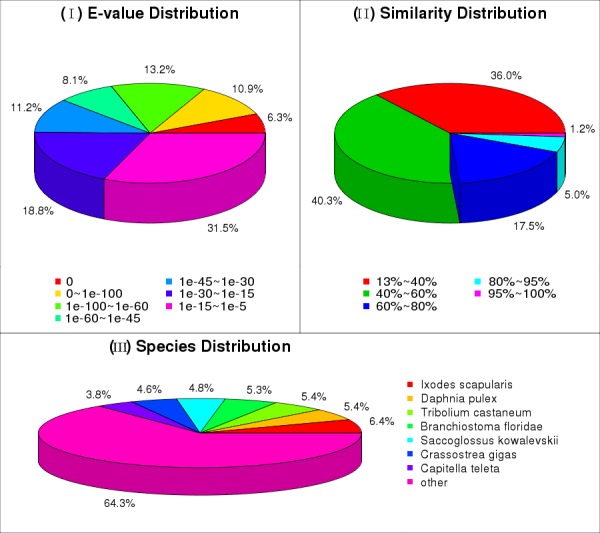
E-value, identity and species distribution statistics of the sequences that can find homologs on Nr database

**Figure 20b.**
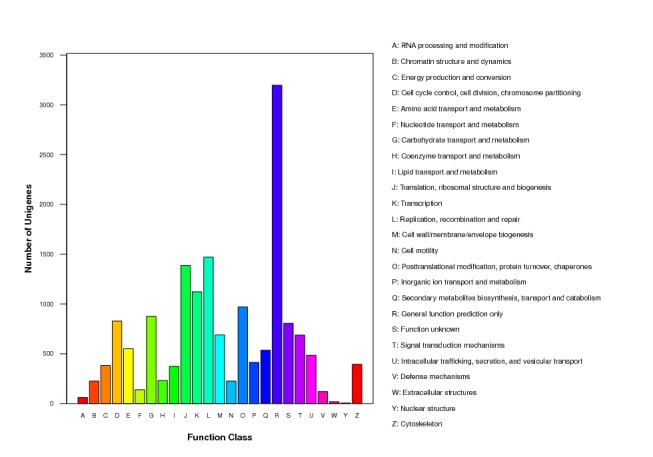
COG functional classification of the transcripts

**Figure 20c.**
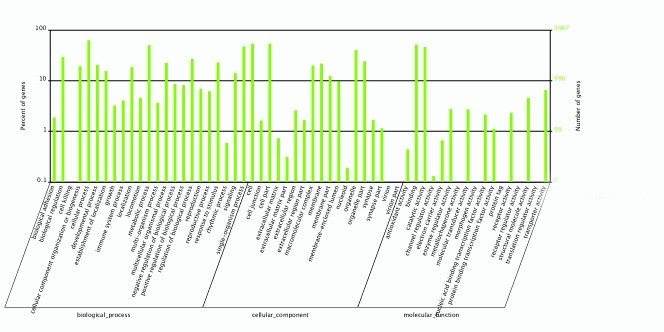
GO categories of the transcripts

**Figure 21.** *Eupolybothrus cavernicolus* Komerički & Stoev sp. n., paratype, 3D model, volume rendering, created with CTVox, virtual rotation and dissection. Movie available at: YouTube.

**Figure 22.** Movie of *Eupolybothrus cavernicolus* Komerički & Stoev sp. n., holotype, filmed ex-situ in a plastic container. Movie available at: YouTube.

**Table 1.** Plectrotaxy of *Eupolybothrus cavernicolus* Komerički & Stoev sp. n., male holotype.

	Ventral					Dorsal				
	Cx	Tr	Pf	F	T	Cx	Tr	Pf	F	T
1			amp	amp	amp			amp	a-p	a
2			amp	amp	amp			amp	a-p	a-p
3			amp	amp	amp			amp	a-p	a-p
4			amp	amp	amp			amp	a-p	a-p
5			amp	amp	amp			amp	a-p	a-p
6			amp	amp	amp			amp	a-p	a-p
7			amp	amp	amp			amp	a-p	a-p
8			amp	amp	amp			amp	a-p	a-p
9			amp	amp	amp			amp	a-p	a-p
10			amp	amp	amp			amp	a-p	a-p
11			amp	amp	amp	a		amp	a-p	a-p
12		m	amp	amp	amp	a		amp	a-p	a-p
13		m	amp	amp	amp	a		amp	a-p	a-p
14		m	amp	am	a	a		amp	a-p	a-p
15	am	m	amp	am	a	a		am	p	-

**Table 2.** Plectrotaxy of *Eupolybothrus cavernicolus* Komerički & Stoev sp. n., female paratype.

	Ventral					Dorsal				
	Cx	Tr	Pf	F	T	Cx	Tr	Pf	F	T
1			amp	amp	amp			amp	a-p	a
2			amp	amp	amp			amp	a-p	a-p
3			amp	amp	amp			amp	a-p	a-p
4			amp	amp	amp			amp	a-p	a-p
5			amp	amp	amp			amp	a-p	a-p
6			amp	amp	amp			amp	a-p	a-p
7			amp	amp	amp			amp	a-p	a-p
8			amp	amp	amp			amp	a-p	a-p
9			amp	amp	amp			amp	a-p	a-p
10			amp	amp	amp			amp	a-p	a-p
11			amp	amp	amp	(a)		amp	a-p	a-p
12		m	amp	amp	amp	(a)		amp	a-p	a-p
13		m	amp	amp	amp	a		amp	a-p	a-p
14	am	m	amp	amp	a	a		amp	a-p	p
15	am	m	amp	am	a	a		amp	p	-

**Table 3.** Interspecific genetic distances (K2P) of *Eupolybothrus* species. Given are the ranges from minimum to maximum values.

		**1**	**2**	**3**	**4**	**5**	**6**	**7**	**8**	**9**	**10**	**11**	**12**
**1**	*Eupolybothrus gloriastygis* BOLD:AAY5019												
**2**	*Eupolybothrus leostygis* BOLD:AAY5071	16.7 - 17.8											
**3**	*Eupolybothrus obrovensis* BOLD:AAY5641	16.2 - 17.0	18.5 - 19.4										
**4**	*Eupolybothrus cavernicolus* BOLD:AAY4900	17.6 - 18.0	14.5 - 15.4	20.8 - 21.2									
**5**	*Eupolybothrus litoralis*	14.7 - 15.1	17.1 - 17.5	17.1 - 17.3	18.0 - 18.1								
**6**	*Eupolybothrus fasciatus*	16.3 - 16.8	18.7 - 19.2	17.5 - 17.7	21.9 - 22.1	13.7							
**7**	*Eupolybothrus tridentinus* GER1 BOLD:AAV7132	17.7 - 18.0	16.7 - 17.3	18.3 - 18.5	17.4 - 17.7	18	18.3						
**8**	*Eupolybothrus tridentinus* GER2 BOLD:AAV7131	17.4 - 17.8	18.6 - 19.1	19.4 - 19.7	18.1 - 18.4	15.7	17.5	10.7					
**9**	*Eupolybothrus transsylvanicus* BOLD:AAJ0488	20.4 - 21.3	20.7 - 21.6	21.4 - 22.1	20.6 - 20.7	16.0 - 16.4	20.4 - 20.8	18.1	19.7 - 20.1				
**10**	*Eupolybothrus kahfi* BOLD:AAY2955	21.9 - 22.5	18.9 - 20.1	21.6 - 21.8	20.0 - 20.2	21	21.7	22.3	21.5	23.2 - 23.6			
**11**	*Eupolybothrus nudicornis* BOLD:AAN2808 BOLD:AAN2810 BOLD:AAN2811	20.1 - 23.2	19.4 - 21.8	21.1 - 24.1	21.2 - 22.7	20.1 - 21.7	21.7 - 22.6	20.7 - 22.4	19.4 - 21.0	21.4 - 22.3	17.2 - 18.8		
**12**	*Eupolybothrus grossipes* BOLD:AAY7960	19.2 - 19.6	21.0 - 21.9	20.9 - 21.1	24.2 - 24.5	16.6	15.3	20.9	18.9	20.3	22.1	20.7 - 22.1	

## Supplementary files

### Raw data used for COI delineation of the *Eupolybothrus* species

**Authors:** Stoev et al. 2013 **Data type:** genomic The archive contains the following data: 1) fasta-Alignment as the basis for all analyses (.FASTA), 2) mega-file for the calculation of the genetic distances and the NJ tree (.MDSX), 3) NJ-tree in Newick format (.NWK), 4) graph of the TCS Software for the Statistical Parsimony method (.GRAPH) **File:** E_cavernicolus.rar
